# Metabolome and transcriptome association analysis revealed key factors involved in melatonin mediated cadmium-stress tolerance in cotton

**DOI:** 10.3389/fpls.2022.995205

**Published:** 2022-09-20

**Authors:** Ling Li, Xuyu Yan, Juan Li, Xiang Wu, Xiukang Wang

**Affiliations:** Shaanxi Key Laboratory of Chinese Jujube, College of Life Sciences, Yan’an University, Yan’an, China

**Keywords:** *Gossypium hirsutum*, Cd stress, Detoxification mechanism, 2-hydroxymelatonin, RNA-Seq, UPLC-MS/MS

## Abstract

Cadmium (Cd), a non-essential element for plant, is a ubiquitous and highly toxic heavy metal, seriously endangering agricultural production and human health. As a nonedible economic crop, cotton (*Gossypium hirsutum* L.) has great potential in remediation of Cd contaminated soil, but its underlying mechanism is still unknown. Melatonin (MT), as a plant growth regulator, is involved in alleviating Cd toxicity in some plants, but the molecular mechanisms of MT-mediated Cd detoxification in cotton are largely unknown. This study investigated the possible molecular mechanisms of the MT-mediated Cd detoxification in cotton seedlings by comparative transcriptomic and metabolomic analyses. The results showed that the cotton seedlings were dwarfed and the leaves were wilted and yellow under Cd stress. The application of 50 *µ*mol L^−1^ MT significantly increased the superoxide dismutase (SOD) activity and malondialdehyde (MDA) content under Cd stress, but 100 *µ*mol L^−1^ MT significantly decreased SOD activity, while increased ascorbate peroxidase (APX) activity significantly. The addition of 100 *μ*mol L^−1^ MT significantly increased Cd concentration in the shoots and roots under Cd stress. RNA-seq analysis showed that 5573, 7105, 7253, 25, 198, 9 up-regulated and 6644, 7192, 7404, 9, 59, 0 down-regulated differentially expressed genes (DEGs) were identified in the comparisons of CK vs T1, CK vs T2, CK vs T3, T1 vs T2, T1 vs T3 and T2 vs T3, respectively. It was revealed that MT promoted the expression of certain related genes under Cd stress, and the effect of 100 *µ*mol L^−1^ MT was better. Moreover, UPLC-MS/MS widely targeted metabolites analyses showed that 195, 150, 150, 12, 24, 59 up-regulated and 16, 11, 23, 38, 127, 66 down-regulated differentially accumulated metabolites (DAMs) were changed in the CK vs T1, CK vs T2, CK vs T3, T1 vs T2, T1 vs T3 and T2 vs T3, respectively. It was revealed that MT induced the synthesis of alkaloids and flavonoids, and inhibited or reduced the synthesis of lipids, amino acids and their derivatives. The comprehensive analyses of transcriptomic and metabolic data showed that 33 DEGs and 4 DAMs, 46 DEGs and 16 DAMs, and 1 DEGs and 1 DAMs were dominantly involved in the pathways of valine, leucine and isoleucine degradation, ABC transporter, alpha-linolenic acid metabolism, respectively. It was revealed that there were three major mechanisms involved in MT-mediated Cd detoxification in cotton, including the enhancement of antioxidant capacity regulated by APX, flavonoids and alkaloids; accumulation of secondary metabolites related to Cd chelation, such as amino acids and derivatives; and regulation of cadmium ion transportation, such as ABC transporter activation. In conclusion, this study provides new insights into the MT-mediated Cd stress response.

## Highlights

1. Application of 100 *µ*mol L^−1^ MT significantly reduced Cd toxicity and promoted seedling growth.
2. The key factors of melatonin-mediated Cd stress tolerance in cotton were revealed.
3. The expression of certain related genes were promoted and the most of alkaloids and flavonoids were accumulated by the addition of MT under Cd stress.
4. The enhancement of antioxidant capacity, the accumulation of secondary metabolites related to Cd chelation, and regulation of Cd ion transportation, were the three major mechanisms involved in MT-mediated Cd detoxification in cotton.

## Introduction

When heavy metal content in soil exceeds the standard, the abiotic stress will cause irreversible damage to plant growth and development (Li et al., 2021). Inappropriate disposal of industrial wastes, sewage irrigation and excessive use of chemical fertilizers and pesticides in agriculture have caused serious cadmium (Cd) pollution in farmland soils worldwide, posing a serious threat to agricultural production and human health (Qin et al., 2021; Yuvaraj et al., 2021). The polluted soil has the characteristics of long-term, cumulative, hidden and irreversibility, so it cannot be repaired and utilized in a short time only by its self-purification ability (Zhang et al., 2021c). Cotton (*Gossypium hirsutum* L.) is a non-edible cash crop with strong tolerance to Cd stress. Although Cd stress can adversely affect the growth and development of cotton, it has its own adaptation and protection mechanism, such as large biomass, strong accumulation capacity, and cotton fiber as the main product does not enter the food chain (Zhu et al., 2020; Wang et al., 2021). Therefore, it has a unique advantage in considering fiber production and soil remediation for Cd contamination.

Melatonin (MT), also known as the pineal gland, is an indoleamine that is vital to life (Rezzani et al., 2020). The content of MT is low in plants, but it can alleviate the damage caused by abiotic stress (such as heavy metals, saline ions, low temperature, drought, bacteria, pests and other biological stress), thus enhancing the resistance of plants to adverse environment (Zhao et al., 2022). It can promote the biosynthesis of glutathione (GSH) and phytochelin under Cd stress, restrict Cd in cell wall and vacuole, and reduce the mobility of Cd in cells by pre-treating the roots of seedlings with MT (Liu et al., 2021b). The plant height, biomass, root growth, GSH content, and the activities of ascorbate peroxidase (APX) and superoxide dismutase (SOD) increased in wheat seedlings, and hydrogen peroxide (H_2_O_2_) content was significantly reduced, then the toxic effects of Cd were greatly reduced by addition of MT (Ni et al., 2018). Therefore, as a new plant growth regulator and biostimulant, melatonin can effectively alleviate Cd toxicity to plants and improve the tolerance to Cd of plants. This is related to the N-acetyl group and 5-methyl group in the chemical structure of MT, which is highly lipophilic and hydrophilic, binds specifically to receptors, and reacts with hydroxyl radicals and peroxy radicals (Arnao & Hernández-Ruiz, 2015). According to the different metabolic pathways of MT, it can be mainly divided into 6-hydroxymelatonin (6-OHMT) and 2-hydroxymelatonin (2-OHMT). 6-hydroxymelatonin is considered to be the most important metabolite of animal MT (Hardeland, 2015). 2-hydroxymelatonin is one of the important metabolites produced by the interaction of melatonin and oxygenates, and is the most important metabolite of plant MT (Byeon et al., 2015; Shah et al., 2020a). Studies have shown that priming seeds with 2-OHMT enhanced photosynthetic rate, water content and gas exchange properties, and also alleviated Cd stress in *Cucumis sativus* seedlings by enhancing non-enzymatic antioxidant accumulation and gene expression (Shah et al., 2020a; Shah et al., 2020b). However, the interactions between Cd stress and MT stress, as well as the molecular and metabolic regulatory mechanisms of MT-mediated Cd stress relief, remain unclear.

Transcriptome analysis provides an important basis for systematically revealing the mechanism of gene expression and transcriptional regulation in different environments (Jiang et al., 2019). Many important studies on transcriptome analysis of plants under Cd stress have been reported (Zhu et al., 2018; Chen et al., 2020; Wang et al., 2022a). Expression of *SaZIP1* in the root and shoot of *Sedum alfredii.* H was significantly induced by Cd stress, and the expression level of Cd high enriched ecotypes was nearly 100 times than that of non-Cd high enriched ecotypes (Gao et al., 2013). The differentially expressed genes (DEGs) of cherry tomato under Cd stress were mainly involved in plant hormone signal transduction, antioxidant enzymes, cell wall biosynthesis, and metal transportation (Hussain et al., 2016). The up-regulated expression of Cd transporter genes *HMA5*, *NRAMP6*, *CAX3*, *ABCC3* and *PDR1* in the leaves of cherry tomato can transport active Cd from the exoplasm to vacuoles (Su et al., 2021). Moreover, DEGs of kenaf were mainly involved in carbohydrate metabolism, replication and repair, signal transduction, transport and catabolism under Cd stress (Chen et al., 2020). There were some key genes that encoded specific Cd transporters, defense systems and highly expressed proteins, thus enhancing Cd tolerance in hemp (Huang et al., 2019). Melatonin accumulation induced by HsfA1a could improve the tolerance of tomato to Cd, while MT partially up-regulated the expression of heat shock proteins (HSPs) and protected the plasma membrane and intracellular proteins of tomato (Cai et al., 2017).

In recent years, metabolomics technology has attracted extensive attention because of its ability to directly reflect plant phenotypic changes and regulate plant gene transcription and protein expression (Arbona et al., 2013; Li & Gaquerel, 2021). Many important studies on metabolomic analysis of plants under Cd stress have been reported. HPLC Q-Exactive technology was used to identify changes in the content of 74 differentially accumulated metabolites (DAMs) in Japanese rice (*Oryza sativa* var. *Japonica*) under Cd stress, which involved in the pathways of amino acid metabolism, purine metabolism, carbon metabolism and glycerolipid metabolism (Navarro-Reig et al., 2017). A highly developed root system in the low-Cd accumulation rice variety (TY816) reduced Cd uptake by up-regulating lipids and fatty acids, while the high-Cd accumulation cultivar (JY841) responded to Cd-induced oxidative stress by up-regulating phenethyl alcohol glycosides (Liu et al., 2021a). Most carbohydrates and amino acids are down-regulated as Cd increases, especially L-cysteine, and inositol is up-regulated as Cd increases to prevent Cd toxicity (Jiang et al., 2020). Simultaneously, 13-(S) -hydroperoxy-9(Z), 11(E), 15(Z) -octadecatrienoic acid was activated as an organic acid, which was related to the metabolism of α-linolenic acid and the production of jasmonic acid (Zeng et al., 2021). The 12 significantly DAMs of *Amaranthus hypochondriacus* under Cd stress were highly linearly correlated with phytochelatins (PCs), which involved in the pathways of valine (Val), leucine (Leu) and isoleucine (Ile) biosynthesis, alanine (Ala), aspartic acid (Asp) and glutamate (Glu) metabolism, and arginine (Arg) and proline (Pro) metabolism (Xie et al., 2019).

Integrated metabolomic and transcriptomic network analyses provide a great opportunity to elucidate the complex response process under environmental stress. It was found several DEGs and DAMs in *Solanum nigrum* L. under Cd stress, including laccase, peroxidase, D-fructose, and cellobiose etc., were related to cell wall biosynthesis and Cd detoxification (Wang et al., 2022a). Study on chickpeas exposed to chromium, Cd and arsenic found that the DEGs of haloacid dehydrogenase, cinnamoyl CoA reductase, F-box protein, GDSL esterase lipase, cellulose synthase, β-glucosidase 13 and isoflavone hydroxylase were significantly enriched, and regulated the pathways of riboflavin metabolism, phenyl propanoid biosynthesis, amino acid biosynthesis, isoflavonoid biosynthesis and indole alkaloid biosynthesis (Yadav et al., 2019). Up to now, the molecular regulation mechanism of plants in response to heavy metal stress is very complicated, so it is very effective to conduct in-depth research on the functioning of a series of DAMs and the corresponding DEGs using the combination of transcriptomic and metabolomic methods.

In this study, different concentrations of MT were added at 200 *µ*mol L^−1^ Cd concentration to analyze the alleviating effect of exogenous MT on Cd toxicity of cotton seedlings. We analyzed phenotypic and physiological responses of cotton seedlings using Illumina high-throughput sequencing technology, and analyzed the changes of metabolites and metabolic pathways using UPLC-MS/MS. Combined with transcriptome and metabolome analysis, the regulatory network of MT-mediated Cd stress in cotton was elucidated and the underlying mechanism was revealed. The purpose of this study was to provide theoretical basis for phytoremediation of heavy metal contaminated soil.

## Materials and methods

### Plant materials and treatments

The cotton variety CCRI 45 was used as the experimental material to study the effect of MT under Cd stress and further remediate Cd-contaminated soil, it is a high-yield and insect-resistant variety selected by Institute of Cotton Research of CAAS with better tolerance to Cd stress. The cotton seeds were surface-sterilized with 3% H_2_O_2_ for 10 min and then washed with tap water three times. After soaking the sterilized seeds in water at 30 °C for 2 h, the seeds were evenly placed in mixed soil at a ratio of vermiculite to organic fertilizer to soil of 2:1:3 in the plastic pots (50 cm length × 25 cm width), and maintained at 28°C and 70% relative humidity in a darkroom. After seed germination, the seedlings were cultured for 14 h at 28 °C under a light intensity of 400 *µ*mol m^−2^ s^−1^ and 10 h at 18 °C under dark conditions every day, and the relative humidity was maintained at 70%.

When the seedlings grew to 2 weeks, the robust and relatively uniform plants were selected and transferred to nutrient solution. The formulation of the nutrient solution (mg L^−1^) referred to Wu et al. (2004). The nutrient solution, with a pH of 6.5± 0.1, was placed in 5L black plastic bucket (20 cm diameter × 20 cm depth). There were 4 holes in the disc cover of each bucket, and the cotton seedlings were fixed in the holes with a sponge, and 3 plants were fixed in each hole. The nutrient solution changed once a week and ventilated with a pump.

The cotton seedlings treated with 200 *µ*mol L^−1^ CdCl_2_ H_2_O after transplanting for one week, and the Cd concentration was screened by our laboratory through more than 10 years of experiments. 2-hydroxymelatonin (CAS: 73-31-4) was used in this experiment, which was purchased from Shanghai Aladdin Biochemical Technology Co., Ltd. The melatonin solutions were prepared by dissolving the solute in ethanol followed by dilution with Milli-Q water [ethanol/water (v/v) =1/10000] (Li et al., 2017). Add different concentrations of MT the next day after Cd treatment. Control plants were grown in the same nutrient solution without Cd. 4 treatments were designed in the experiment, that is 0 (CK), 200 *µ*mol L^−1^ Cd (T1), 50 *µ*mol L^−1^ MT+200 *µ*mol L^−1^ Cd (T2), 100 *µ*mol L^−1^ MT+200 *µ*mol L^−1^ Cd (T3), and each treatment was repeated 3 times, 12 plants per pot. After each treatment for 3 days, the cotton seedlings changed obviously, 1 g of fresh leaves of each sample were collected for determination of antioxidative enzyme and lipid peroxides, and 2 g of fresh leaves were wrapped with tin foil, then immediately frozen in the liquid nitrogen and stored at -80°C prior to RNA-Seq and metabolite extraction.

### Determination of antioxidative enzyme and lipid peroxides

After each treatment for 3 days, 1 g of fresh leaves from plants undergoing different treatments was placed into a mortar, and 10 mL of 50 mmol L^−1^ phosphate buffer (pH 7.8, containing 0.1 mmol L^−1^ EDTA) and a small amount of quartz sand were added and ground into a homogenate in an ice bath. The sample was transferred to a 10 mL centrifuge tube and centrifuged at 12000 g at 4 °C for 20 min. The supernatant was stored in a refrigerator at 4 °C prior to the determination of antioxidant enzyme activity and malondialdehyde (MDA) content. The activity of SOD was determined by the nitrotetrazolium blue chloride (NBT) method at 560 nm (Beyer & Fridovich, 1987). Peroxidase (POD) activity was measured using guaiacol oxidation at 470 nm (Pütter, 1974). Ascorbate peroxidase (APX) activity was determined according to the method of (Chen & Asada, 1989). The MDA content represented the level of lipid peroxidation products and was determined by the reaction of 2-thiobarbituric acid (TBA) (Heath & Packer, 1968).

### Determination of Cd concentration

The Cd concentration was determined using Inductively Coupled Plasma-Mass Spectrometry (ICP-MS) (7500a, Agilent). Briefly, the shoots and roots were collected separately, dried at 80°C to constant weight, then ground to a fine powder and passed through a 120-mesh sieve. 0.1 g of sample was weighed accurately into quartz digestion cup, then digested for 4-5 hours by adding a mixture of 4-5 ml HNO_3_ and 0.5 ml H_2_O_2_. The digested sample solution was transferred to a 50 mL volumetric flask to volume, and Cd was quantified using ICP Mass Spectrograph.

### Transcriptome sequencing and analysis

Total RNA was extracted from the leaves of 12 cotton samples using Trizol reagent (Invitrogen, Carlsbad, CA, USA). Three biological replicates were performed. The concentration, purity and integrity of RNA samples were detected by Qubit 2.0 Flurometer, NanoPhotometer spectrophotometer and Agilent 2100 bioanalyzer. The cDNA library construction and sequencing were performed by Wuhan Met Ware Biotechnology Co., Ltd. (www.metware.cn) using the Illumina high-throughput sequencing platform. Clean reads were obtained by referring to the method of (Chen et al., 2018), and mapped to the cotton reference genome (*Gossypium hirsutum.* L) using HISAT v2.1.0 (Kim et al., 2015). The gene alignment and the FPKM of each gene based on the gene length were calculated using Feature Counts v1.6.2 (Liao et al., 2014). The DEGs were analyzed using DESeq2 v1.22.1, which were identified according to |log2fold change|≥1 and false discovery rate (FDR)<0.05 (Love et al., 2014). And in order to infer the function of DEGs, BLAST software and KOBAS 2.0 software were used for KEGG pathway analysis.

### Metabolite analysis

Sample freeze-drying, crushing and extraction, and metabolome profiling and data analysis were carried out at Wuhan MetWare Biotechnology Co., Ltd. (www.metware.cn) following their standard procedures (Guo et al., 2019). Three biological replicates were performed. The UPLC-MS/MS system was used for metabolome profiling, which was composed of Ultra Performance Liquid Chromatography (UPLC) and Tandem mass spectrometry (MS/MS). The conditions of UPLC and MS referred to the detailed procedures of Guo et al. (2019). The mass spectrometry software analyst 1.6.3 was used to process the data obtained, and partial least squares-discriminant analysis (PLS-DA) was carried out to DAMs, which satisfying variable importance of the projection (VIP) ≥1 and |log2fold change| ≥1 were defined as DAMs. The functional annotation of DAMs was carried out based on KEGG and analyzed the relevant pathways, their significance was determined by the P value of the hypergeometric test.

### Integrated analysis between DEGs and DAMs

In order to establish the data relationship, the transcriptome and metabolome data were normalized and statistically analyzed, key genes, metabolites and metabolic pathways were screened out by functional analysis, metabolic pathway enrichment and correlation analysis. The Pearson correlation analysis between DEGs and DAMs was using the cor function of R language with normalized data. The analysis of correlation and KEGG enrichment was performed using DEGs and DAMs Pearson Correlation Coefficient (PCC)| ≥0.8). And a network diagram of DEGs and DEMs was drawn to analyze the relationship between genes and metabolites.

### Data analysis

Data collation, calculation and histogram drawing using Microsoft Excel 2010, variance analysis using SPSS 20.0, each data was expressed in the form of mean ± MS, and *t*-test analysis was performed at the *P*<0.05 level.

## Results

### Phenotype and physiology responses of cotton seedling

The phenotype of cotton seedlings was investigated and physiological responses of leaves were analyzed after 3 days of each treatment. Compared with the control (CK), 200 *µ*mol L^−1^ Cd significantly inhibited the growth of cotton seedlings, the plants were short and the leaves were yellow and withered (T1). The Cd toxicity can be reduced by adding MT, and the effect of 100 *µ*mol L^−1^ MT was significantly better than that of 50 *µ*mol L^−1^ MT, but the growth of cotton seedling plants is still worse than that of CK (**Figure S1-A**). The activities of SOD, POD and APX, and MDA content in cotton seedling leaves were significantly higher under 200 *µ*mol L^−1^ Cd treatment (T1) than that of the control (CK). Compared with the Cd treatment alone (T1), the SOD activity and MDA content increased significantly after adding 50 *µ*mol L^−1^ MT (T2), while POD and APX decreased significantly. After adding 100 *µ*mol L^−1^ MT (T3), the SOD activity decreased significantly, the POD activity and MDA content also decreased, but the difference was not obvious, while the APX activity increased significantly (**Figure S1B**). The results preliminarily showed that 100 *µ*mol L^−1^ MT was more effective than 50 *µ*mol L^−1^ MT in reducing the toxicity of Cd.

### Cd concentration in cotton shoots and roots

The concentration of Cd in shoots and roots of cotton seedlings determined after under Cd stress and adding melatonin (**Figure S2**). The Cd concentration in roots was significantly higher than in shoots, showing that more Cd accumulated by root absorption under the same conditions. Compared to the control, Cd concentration was significantly increased in shoots and roots under 200 *µ*mol L^−1^ Cd (T1). Compared with the Cd treatment alone (T1), the addition of MT significantly increased Cd concentration, but the effect between 50 *µ*mol L^−1^ MT (T2) and 100 *µ*mol L^−1^ MT (T3) was not obvious in the shoots. In roots, Cd concentration was significantly increased by adding 100 *µ*mol L^−1^ MT (T3) compared with Cd treatment alone (T1), but decreased by adding 50 *µ*mol L^−1^ MT (T2), and the difference was not significant.

### Transcriptome analysis

The transcriptome responses of leaves were investigated by RNA-seq after 3 days of each treatment. This study obtained a total of 80.39 Gb clean data, and the clean data of each sample reached 6 Gb. The GC content was 43.00%-44.18%, and the Q30 was >94.04% (**Figure S3**). The comparison efficiency was 97.64%-98.06% between the reads of each sample and the reference genome. The clean data generated in RNA-seq were submitted to NCBI (PRJNA818420). Based on the comparison results, the mapped reads were assembled and compared with the published annotation information of the upland cotton genome, alternative splicing analysis and gene structure optimization were performed, and a total of 2688 new genes were discovered. The DEGs in different samples were screened and then performed functional annotation and enrichment analysis.

**Figure 1:**
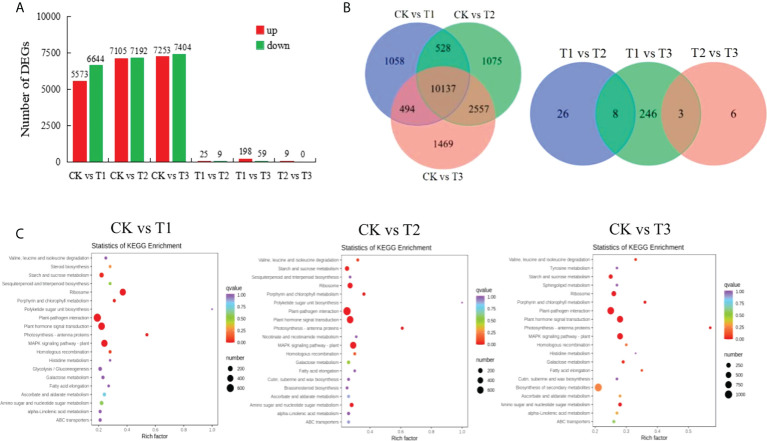
The number distribution, venn diagram and KEGG pathway enrichment map of DEGs in different sample group. **(A)**, The number distribution of DEGs among CK vs T1, CK vs T2, CK vs T3, T1 vs T2, T1 vs T3, T2 vs T3; **(B)**, Venn diagram of DEGs among CK vs T1, CK vs T2, CK vs T3, T1 vs T2, T1 vs T3, T2 vs T3; **(C)**, KEGG pathway enrichment map of DEGs among CK vs T1, CK vs T2, CK vs T3. CK refers to the control, T1 refers to 200 *µ*mol L^−1^ Cd treatment, T2 refers to the treatment of 50 *µ*mol L^−1^ MT+200 *µ*mol L^−1^ Cd, T3 refers to the treatment of 100 *µ*mol L^−1^ MT+200 *µ*mol L^−1^ Cd.

A total of 41471 DEGs were screened, of which 20163 were up-regulated and 21,308 were down-regulated (**Figure 1A**). The numbers of DEGs were significantly higher in CK vs T1, CK vs T2, CK vs T3 than that in T1 vs T2, T1 vs T3, T2 vs T3. It indicated that the effects on the gene expression were significant in cotton seedlings under Cd treatment or the addition of MT. And certain genes were promoted by adding MT under Cd stress, especially 100 *µ*mol L^−1^ MT has a better effect. There were 3 DEGs in CK vs T1, CK vs T2, CK vs T3, T1 vs T2, T1 vs T3, which were *novel.1853*, *gene-LOC107922917*, and *novel.79*; 10137 DEGs in CK vs T1, CK vs T2, CK vs T3; and 8 DEGs in T1 vs T2, T1 vs T3, which were *novel.1853*, *gene-LOC107922917*, *novel.79*, *novel.1760*, *novel.1572*, *novel.2101*, *gene-LOC107954558*, *gene-LOC107894809*; 3 DEGs in T1 vs T3, T2 vs T3, which were *gene-LOC121202834*, *gene-LOC107912207*, *gene-LOC107932195* (**Figure 1B**). Only 9 DEGs in T2 vs T3, and all of them were up-regulated, it can be explained why the detoxification effect of Cd by adding 100 *µ*mol L^−1^ MT was better than that of 50 *µ*mol L^−1^ MT. Therefore, these DEGs can be used as the source of candidate genes for responding to Cd stress and screening MT concentration.

The KEGG enrichment analysis indicated that DEGs of CK vs T1, CK vs T2, CK vs T3 were significantly enriched (P-value<0.01) in the pathways of photosynthesis-antenna proteins, ribosome, MAPK signaling pathway-plant, plant-pathogen interaction, plant hormone signal transduction, porphyrin and chlorophyll metabolism, amino sugar and nucleotide sugar metabolism, starch and sucrose metabolism, galactose metabolism, valine, leucine and isoleucine degradation (**Figure 1C**). No DEGs significantly enriched in T1 vs T2, T1 vs T3, T2 vs T3 (P-value<0.01). However, the pathways of alpha-Linolenic acid metabolism, MAPK signaling pathway-plant, and proteasome were significantly enriched (P-value<0.05) by DEGs in T1 vs T3.

### Metabonomic analysis

**Figure 2:**
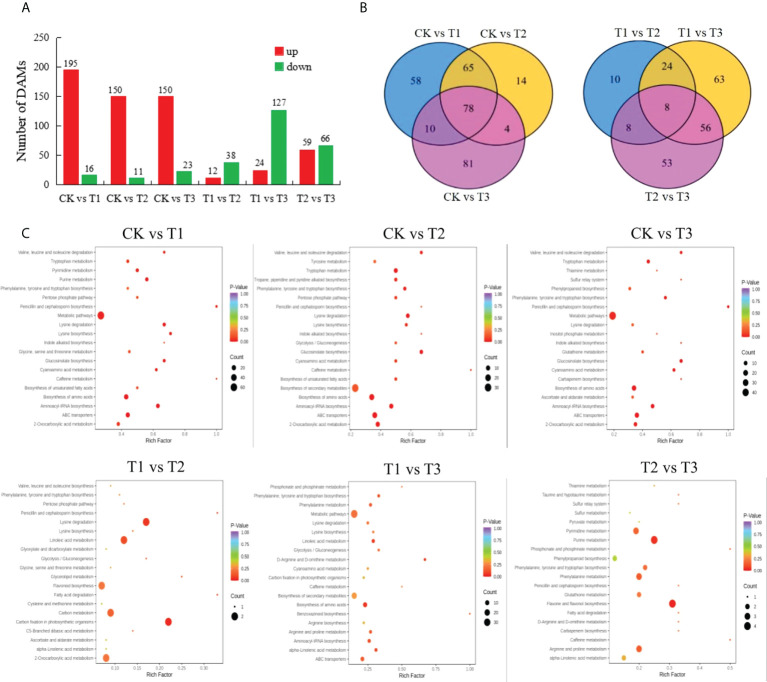
The number distribution, venn diagram and KEGG pathway enrichment map of DAMs in different sample group. **(A)**, The number distribution of DAMs among CK vs T1, CK vs T2, CK vs T3, T1 vs T2, T1 vs T3, T2 vs T3; **(B)**, Venn diagram of DAMs among CK vs T1, CK vs T2, CK vs T3, T1 vs T2, T1 vs T3, T2 vs T3; **(C)**, KEGG pathway enrichment map of DAMs among CK vs T1, CK vs T2, CK vs T3, T1 vs T2, T1 vs T3, T2 vs T3. CK refers to the control, T1 refers to 200 *µ*mol L^−1^ Cd treatment, T2 refers to the treatment of 50 *µ*mol L^−1^ MT+200 *µ*mol L^−1^ Cd, T3 refers to the treatment of 100 *µ*mol L^−1^ MT+200 *µ*mol L^−1^ Cd.

The metabolome responses of cotton seedlings leaves were investigated using UPLC-MS/MS after 3 days of each treatment. The results showed that a total of 1019 metabolites were identified from the 6 treatment combinations. The numbers of DAMs were significantly higher in CK vs T1, CK vs T2, CK vs T3 than that of T1 vs T2, T1 vs T3, T2 vs T3, which was basically the same as the change trend of DEGs, but the changes of DAMs were not obvious than that of DEGs (**Figure 2A**). The results also indicated that certain metabolites were stimulated by Cd stress, and the down-regulated of DAMs were reduced by the addition of MT under Cd stress, especially 100 *µ*mol L^−1^ MT. The numbers of up-regulated DAMs were significantly higher than that of down-regulated DAMs in CK vs T1, CK vs T2, CK vs T3, on the contrary, the up-regulated DAMs were significantly lower than that of down-regulated DAMs in T1 vs T2, T1 vs T3, T2 vs T3. In particular, 100 *µ*mol L^−1^ MT significantly caused the down-regulated of a large number of DAMs under Cd stress.

From the Venn diagram (**Figure 2B**), it can be seen that there were 5 DAMs in CK vs T1, CK vs T2, CK vs T3, T1 vs T2, T1 vs T3, which were DL-2-aminoadipic acid, scoparone, solasodiene, LysoPC 18:3(2n isomer), genistein-7-O-(6’’-malonyl) glucoside. Scoparone and genistein-7-O- (6’’-malonyl) glucoside were up-regulated in all combinations, and DL-2-aminoadipic acid, solasodiene and LysoPC 18:3 (2n isomer) were up-regulated in CK vs T1, CK vs T2, CK vs T3, while down-regulated in T1 vs T2 and T1 vs T3. And there were 78 DAMs in CK vs T1, CK vs T2, CK vs T3, 8 DAMs in T1 vs T2, T1 vs T3, T2 vs T3. Among the 8 DAMs, D-Glucurono-6,3-lactone was up-regulated, 9-Hydroxy-10,12,15-octadecatrienoic acid, 13-KODE; (9Z,11E)-13-Oxooctadeca-9,11-dienoic acid (13-KODE), 9,12,13-Trihydroxy-10,15-octadecadienoic acid, LysoPC 14:0, LysoPC 16:0(2n isomer) and LysoPC 19:1 were all lipids and down-regulated in T1 vs T2, T1 vs T3, T2 vs T3. And 24,30-Dihydroxy-12(13)-enolupinol was down-regulated in T1 vs T2, while up-regulated in T1 vs T3 and T2 vs T3. Therefore, these DAMs can be used as candidate metabolites for responding to Cd stress and screening MT concentration.

The analysis of KEGG enrichment was performed on the all-treatment combinations, only the pathway of aminoacyl-tRNA biosynthesis was significantly enriched (P-value<0.05) by DAMs in CK vs T1 (**Figure 2C**). Therefore, the KEGG enrichment analysis was conducted at P-value<0.1, it found that the pathways of aminoacyl-tRNA biosynthesis, lysine degradation, biosynthesis of amino acids, ABC transporters, purine metabolism, glucosinolate biosynthesis, lysine biosynthesis, penicillin and cephalosporin biosynthesis, pyrimidine metabolism, cyanoamino acid metabolism, valine, leucine and isoleucine degradation, tryptophan metabolism, 2-Oxocarboxylic acid metabolism, phenylalanine, tyrosine and tryptophan biosynthesis, tropane, piperidine and pyridine alkaloid biosynthesis, caffeine metabolism, pentose phosphate pathway, biosynthesis of unsaturated fatty acids, carbon fixation in photosynthetic organisms, D-Arginine and D-ornithine metabolism, flavone and flavonol biosynthesis were significantly enriched by DAMs in the all treatment combinations.

The top 10 DAMs of up-regulated and down-regulated in the all-treatment combinations were listed in **Table 1** according to the value of log2 fold change. It can be seen that the up-regulated DAMs were isoquinoline, α-Solasonine, 4,8-Dihydroxyquinoline-2-carboxylic acid, 3-Indoleacetonitrile, 3-Indolepropionic acid, LysoPE 15:1 and DL-2-Aminoadipic acid in CK vs T1, CK vs T2, CK vs T3, which contained 5 alkaloids, 1 organic acid and 1 lipid; the down-regulated DAMs were N-Acetyl-L-glutamic acid, 3-Guanidinopropionic acid and 1-(9Z-Octadecenoyl)-2-(9-oxo-nonanoyl)-sn-glycero-3-phosphocholine, which were 1 amino acid and its derivatives, 1 organic acid and 1 lipid respectively. The up-regulated DAMs were chrysin-5-O-glucoside, genistein-7-O-(6’’-malonyl) glucoside, acetryptine, scoparone, D-glucurono-6,3-lactone, erythorbic acid (isoascorbic acid) in T1 vs T2 and T1 vs T3, which were 2 flavonoids, 1 alkaloid, 1 lignans and coumarins, 2 other kinds; the down-regulated DAMs were 9,12,13-Trihydroxy-10,15-octadecadienoic acid, LysoPC 16:0, LysoPC 17:0, LysoPC 20:0, all of which were lipids. And up-regulated DAMs were esculetin-7-O-glucoside, esculin (6,7-DihydroxyCoumarin-6-glucoside) and 24,30-Dihydroxy-12(13)- enolupinol in T1 vs T3 and T2 vs T3, which contained 2 lignans and coumarins, 1 terpene; the down-regulated DAMs was L-Alanyl-L-Phenylalanine, which belonged to amino acid and derivatives. So, it can be seen that DAMs with large up-regulation were mainly alkaloids, while that with large down-regulation were riched in types and relatively more lipids.

**Table 1:** Top ten DAMs (up or down regulated) among different sample groups.

Sample group	Up-regulated compounds [Log_2_ (FC)]	Down-regulated compounds [Log_2_ (FC)]
CK vs T1	Isoquinoline (14.92), α-Solasonine (14.55), 3-Indoleacetonitrile (12.81), 3-Indolepropionic acid (12.70), 4,8-Dihydroxyquinoline-2-carboxylic acid (12.62), LysoPE 15:1 (10.08), DL-2-Aminoadipic acid (5.98), 9,12,13-Trihydroxy-10,15-octadecadienoic acid (5.97), Indole (5.13), Methoxyindoleacetic acid (4.92)	N-Acetyl-L-glutamic acid (-3.75), D-Glucurono-6,3-lactone (-2.65), Erythorbic Acid (Isoascorbic Acid) (-2.34), 1-(9Z-Octadecenoyl)-2-(9-oxo-nonanoyl)-sn-glycero-3-phosphocholine (-2.07), O-Acetylserine (-1.65), Guanosine 5’-monophosphate (-1.25), Inosine 5’-monophosphate (-1.19), 3-Guanidinopropionic acid (-1.19), Curcumenol (-1.15), Pinoresinol-4-O-(6’’-acetyl) glucoside (-1.09)
CK vs T2	Chrysin-5-O-glucoside (15.81), α-Solasonine (15.30), Isoquinoline (14.49), 4,8-Dihydroxyquinoline-2-carboxylic acid (12.23), 3-Indoleacetonitrile (12.10), 3-Indolepropionic acid (11.77), Genistein-7-O-(6’’-malonyl) glucoside (9.51), LysoPE 15:1 (8.95), DL-2-Aminoadipic acid (4.91), Cytarabine (4.72)	N-Acetyl-L-glutamic acid (-3.75), O-Acetylserine (-1.96), 1-(9Z-Octadecenoyl)-2-(9-oxo-nonanoyl)-sn-glycero-3-phosphocholine (-1.77), 2-Deoxyribose-5’-phosphate (-1.72), D-Fructose-1,6-biphosphate (-1.55), 2-Deoxyribose-1-phosphate (-1.53), D-Sedoheptuiose 7-phosphate (-1.30), Iminodiacetic acid (-1.17), 3-Guanidinopropionic acid (-1.17), 6-Hydroxydaidzein (-1.10)
CK vs T3	Chrysin-5-O-glucoside (15.88), Isoquinoline (14.25), α-Solasonine (13.21), 4,8-Dihydroxyquinoline-2-carboxylic acid (12.63), 24,30-Dihydroxy-12(13)-enolupinol (12.46), 3-Indoleacetonitrile (12.22), 3-Indolepropionic acid (11.77), Genistein-7-O-(6’’-malonyl) glucoside (9.56), LysoPE 15:1 (9.25), DL-2-Aminoadipic acid (4.68)	N-Acetyl-L-glutamic acid (-4.19), Cyclic 3’,5’-Adenylic acid (-2.00), 3-Guanidinopropionic acid (-1.98), 2-Hydroxycinnamic acid (-1.77), γ-Glu-Cys (-1.76), 2-(4-Hydroxybenzyl)-4-(methoxymethyl) phenol (-1.59), N-Acetylcadaverine (-1.59), 1-(9Z-Octadecenoyl)-2-(9-oxo-nonanoyl)-sn-glycero-3-phosphocholine (-1.59), Ferulic acid (-1.56), Glutathione reduced form (-1.52)
T1 vs T2	Chrysin-5-O-glucoside (15.81), Genistein-7-O-(6’’-malonyl) glucoside (7.65), Acetryptine (2.43), Scoparone (2.19), Erythorbic Acid (Isoascorbic Acid) (1.99), D-Glucurono-6,3-lactone (1.87), Luteolin-6-C-glucoside (1.49), Levopimaric acid (1.40), Pinoresinol-4-O-(6’’-acetyl) glucoside (1.37), Tectochrysin (1.20)	24,30-Dihydroxy-12(13)-enolupinol (-7.50), 9,12,13-Trihydroxy-10,15-octadecadienoic acid (-3.93), LysoPC 17:0(2n isomer) (-2.54), Caffeoyl(p-Hydroxybenzoyl) tartaric acid (-2.39), LysoPC 20:0 (-2.32), LysoPC 17:0 (-2.07), LysoPC 16:0(2n isomer) (-1.87), LysoPC 15:0(2n isomer) (-1.67), LysoPC 20:2(2n isomer) (-1.64), 15(R)-Hydroxylinoleic Acid (-1.54)
T1 vs T3	Chrysin-5-O-glucoside (15.88), Genistein-7-O-(6’’-malonyl) glucoside (7.69), 24,30-Dihydroxy-12(13)-enolupinol (4.96), Erythorbic Acid (Isoascorbic Acid) (3.74), D-Glucurono-6,3-lactone (3.73), Acetryptine (2.93), Genistein-8-C-glucoside (2.40), Esculetin-7-O-glucoside (1.67), Esculin (6,7-DihydroxyCoumarin-6-glucoside) (1.57), Scoparone (1.47)	9,12,13-Trihydroxy-10,15-octadecadienoic acid (-5.67), LysoPC 18:0(2n isomer) (-3.61), LysoPC 17:0(2n isomer) (-3.03), 13-KODE; (9Z,11E)-13-Oxooctadeca-9,11-dienoic acid (-2.97), L-Alanyl-L-Phenylalanine (-2.89), LysoPC 16:0(2n isomer) (-2.88), LysoPC 20:0 (-2.86), LysoPC 17:0 (-2.76), LysoPC 19:1 (-2.69), Isopimaric acid (-2.68)
T2 vs T3	24,30-Dihydroxy-12(13)-enolupinol (12.46), Kaempferol-3-O-(6’’-acetyl) glucoside (3.27), Sesquimarocanol B (3.02), Kaempferol-3-O-(6’’-malonyl) galactoside (2.60), Eriodictyol-7-O-(6’’-malonyl) glucoside (2.56), Kaempferol-3-O-(6’’-malonyl) glucoside (2.55), Persicoside (2.53), Esculetin-7-O-glucoside (2.45), 4-Acetamidobutyric acid (2.42), Esculin (6,7-DihydroxyCoumarin-6-glucoside) (2.32)	L-Alanyl-L-Phenylalanine (-3.65), L-Valyl-L-Leucine (-3.55), L-Valyl-L-Phenylalanine (-3.53), L-Leucyl-L-Leucine (-3.29), L-Seryl-L-Isoleucine (-2.74), Uridine (-2.61), L-Leucyl-L-phenylalanine (-2.31), Crotonoside; 2-Hydroxyadenosine (-2.31), 1-Oleoyl-Sn-Glycerol (-2.24), α-Solasonine (-2.09)

### Correlation analysis between DEGs and DAMs

**Figure 3:**
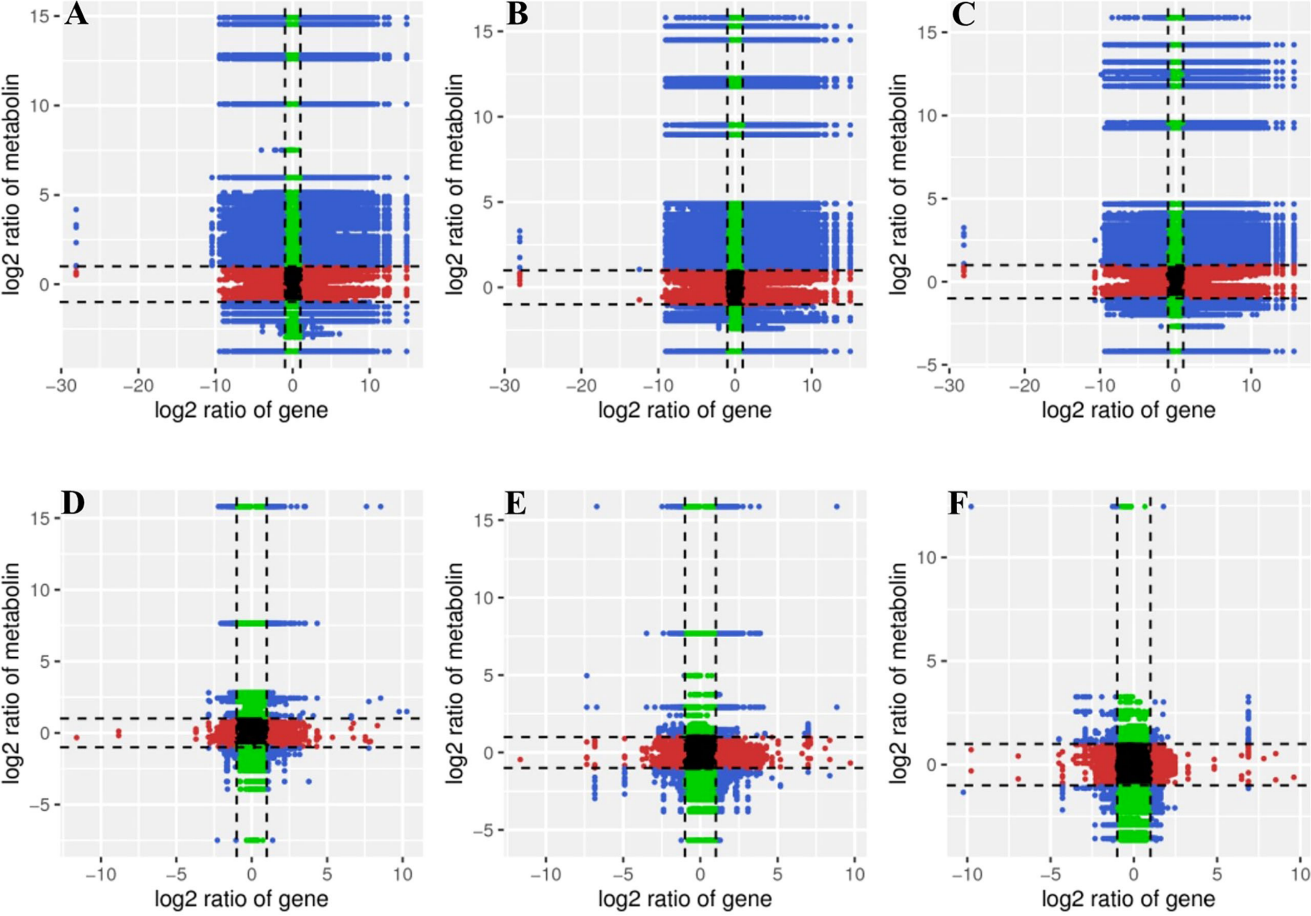
Correlation quadrant diagrams representing association of DEGs and DAMs. **(A)**, CK vs T1; **(B)**, CK vs T2; **(C)**, CK vs T3; **(D)**, T1 vs T2; **(E)**, T1 vs T3; **(F)**, T2 vs T3.

Log2 conversion data for DEGs and DAMs were selected with a Pearson’s correlation coefficient (PCC)>0.8. The nine quadrant diagrams were generated to obtain a systematic view of the variations in DAMs and their corresponding DEGs respond to Cd stress and the addition of MT (**Figure 3**). The black dotted lines divide each graph into 9 quadrants. Unchanged DAMs and unchanged DEGs were displayed in quadrant 5, up-regulated DAMs and up-regulated DEGs were displayed in quadrant 3, down-regulated DAMs and down-regulated DEGs were displayed in quadrant 7, and the DAMs and DEGs shown in quadrant 3 and quadrant 7 were positively correlated and had similar consistent patterns. Up-regulated DAMs and down-regulated DEGs were displayed in quadrant 1, down-regulated DAMs and up-regulated DEGs were displayed in quadrant 9, while the DAMs and DEGs shown in quadrant 1 and quadrant 9 were negatively correlated and had opposite patterns. The unchanged DAMs and up-regulated or down-regulated DEGs, as well as the unchanged DEGs and up-regulated or down-regulated DAMs were displayed in quadrant 2, 4, 6, 8.

As shown in quadrant 3,7, 11430 DEGs corresponding to 219 DAMs in CK vs T1 (**Figure 3A**), 12645 DEGs corresponding to 162 DAMs in CK vs T2 (**Figure 3B**), 12834 DEGs corresponding to 195 DAMs in CK vs T3 (**Figure 3C**), 578 DEGs corresponding to 54 DAMs in T1 vs T2 (**Figure 3D**), 1150 DEGs corresponding to 145 DAMs in T1 vs T3 (**Figure 3E**), 158 DEGs corresponding to 96 DAMs in T2 vs T3 (**Figure 3F**).

### KEGG analysis of DEGs and DAMs

**Figure 4:**
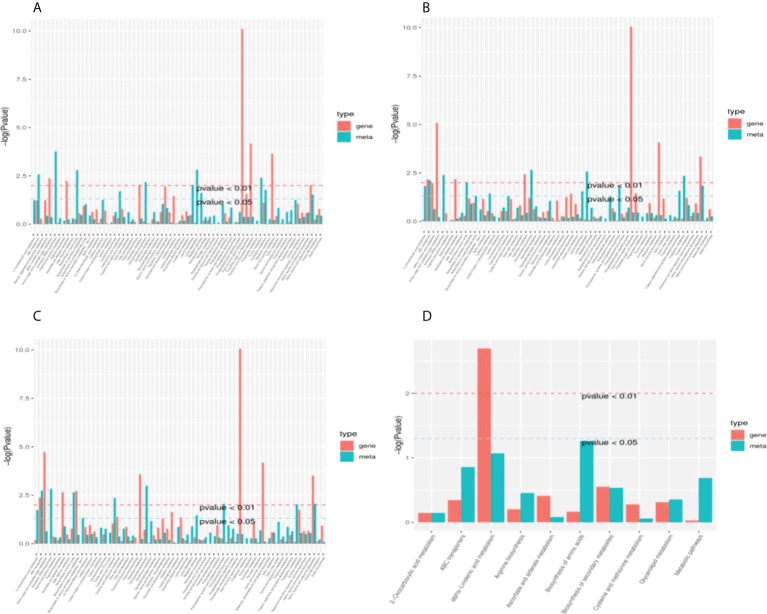
KEGG enrichment analysis pvalue histogram of DEGs and DAMs. **(A)**, CK vs T1; **(B)**, CK vs T2; **(C)**, CK vs T3; **(D)**, T1 vs T3. The abscissa represents metabolic pathways, and the ordinate represents the enriched pvalue of DEGs (red) and DAMs (green), which is represented by -log (Pvalue). The higher the ordinate, the stronger the enrichment degree.

According to the results of KEGG enrichment analysis on DEGs and DAMs of cotton seedling leaves in response to Cd stress and the addition of MT, a histogram was drawn to show the enrichment degree of pathways with DEGs and DAMs simultaneously. The results were shown in **Figure 4**. 5114 DEGs and 328 DAMs were enriched to 68 metabolic pathways in CK vs T1 (**Figure 4A**), 5803 DEGs and 268 DAMs were enriched to 63 metabolic pathways in CK vs T2 (**Figure 4B**), 6105 DEGs and 256 DAMs were enriched to 64 metabolic pathways in CK vs T3 (**Figure 4C**), 8 DEGs and 10 DAMs were enriched to 3 metabolic pathways in T1 vs T2, 43 DEGs and 85 DAMs were enriched to 10 metabolic pathways in T1 vs T3 (**Figure 4D**), 6 DEGs and 33 DAMs were enriched to 4 metabolic pathways in T2 vs T3.

Interestingly, it was found that DEGs and DAMs in CK vs T1 were simultaneously significantly enriched in the pathway of valine, leucine and isoleucine degradation (P value<0.05), which was stimulated by Cd stress. The pathways of valine, leucine and isoleucine degradation and ABC transporter were significantly enriched (P-value<0.05) by DEGs and DAMs simultaneously in CK vs T2 and CK vs T3, it indicated that the pathway of valine, leucine and isoleucine degradation was still active under Cd stress after adding MT, and the ABC transporter are stimulated simultaneously. Only the pathway of alpha-linolenic acid metabolism was significantly enriched (P-value<0.05) by DEGs and DAMs simultaneously in T1 vs T3, it showed that the production of alpha-linolenic acid was promoted by 100 *µ*mol L^−1^ MT in response to Cd stress. There were no significant enrichment pathways in the treatment groups of T1 vs T2 and T2 vs T3, it indicated that no metabolic pathway was obviously stimulate by 50 *µ*mol L^−1^ MT, and the differences on MT concentrations were not obvious for the excitation of metabolic pathways. In conclusion, the three pathways of valine, leucine and isoleucine degradation, ABC transporter, and alpha-linolenic acid metabolism may be the key and important pathways for cotton leaves to respond to Cd stress and melatonin to alleviate Cd toxicity.

### Co-expression network analysis of DEGs and DAMs

**Figure 5:**
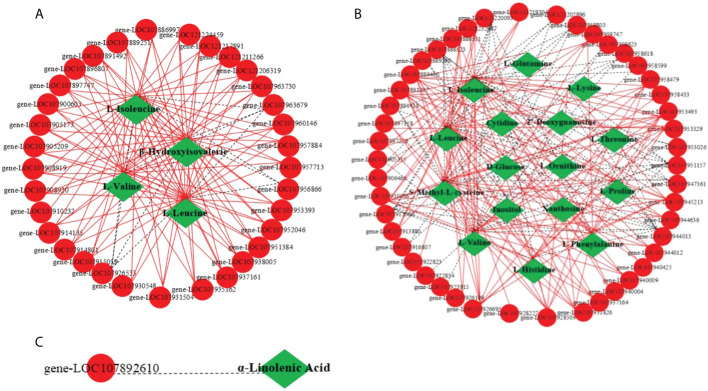
The network analysis of DEGs and DAMs in response to Cd stress and the addition of MT in cotton seedling leaves. **(A)** the correlation network of DEGs and DAMs involved in the pathway of valine, leucine and isoleucine degradation; **(B)** the correlation network of DEGs and DAMs involved in the pathway of ABC transporter; **(C)**, the correlation network of DEGs and DAMs involved in the pathway of alpha-linolenic acid metabolism; ●The red circle represents DEGs; ♦the green square represents DAMs, the solid red line represents positive correlation, and the dashed black line represents negative correlation.

A correlation network diagram of DEGs and DAMs (|PCC|≥0.8, P-value<0.05) was conducted to further analyze the correlation between the DEGs and DAMs involved in the three metabolic pathways of valine, leucine and isoleucine degradation, ABC transporter and alpha-Linolenic acid metabolism (**Figure 5**). The results showed that a total of 33 DEGs and 4 DAMs were involved in the pathway of valine, leucine and isoleucine degradation, and β-hydroxyisovaleric acid was related to pathway. In this pathway, only three genes, *gene-LOC107926533*, *gene-LOC107956866* and *gene-LOC107963679*, were negatively correlated with DAMs, and the others were positively correlated. A total of 46 DEGs and 16 DAMs were involved in the ABC transporter pathway, in which commonly enriched by 11 amino acids (L-isoleucine, L-leucine, S-methyl-L-cysteine, L-valine, L-histidine, L-phenylalanine, L-proline, L-threonine, L-lysine, L-glutamine, L-ornithine) and D-glucose, inositol, cytidine, 2’-Deoxyguanosine, xanthosine. In this pathway, *gene-LOC107959623* and *gene-LOC107889290* were only involved in negative regulation of D-glucose and inositol; *gene-LOC107916807*, *gene-LOC107940004* and *gene-LOC107953026* were only involved in negative regulation of cytidine, 2’-Deoxyguanosine, xanthosine; the other genes were participated in the positive regulation of amino acid metabolism. Only *gene-LOC107892610* was involved in the negative regulation of α-linolenic acid metabolism. Therefore, it indicated that the co-expression of DEGs and DAMs related to the pathways of valine, leucine and isoleucine degradation, ABC transporter, alpha-linolenic acid metabolism can be regulated by MT under Cd stress.

### Comprehensive analysis of related DEGs and DAMs in the pathway of valine, leucine and isoleucine degradation

**Figure 6:**
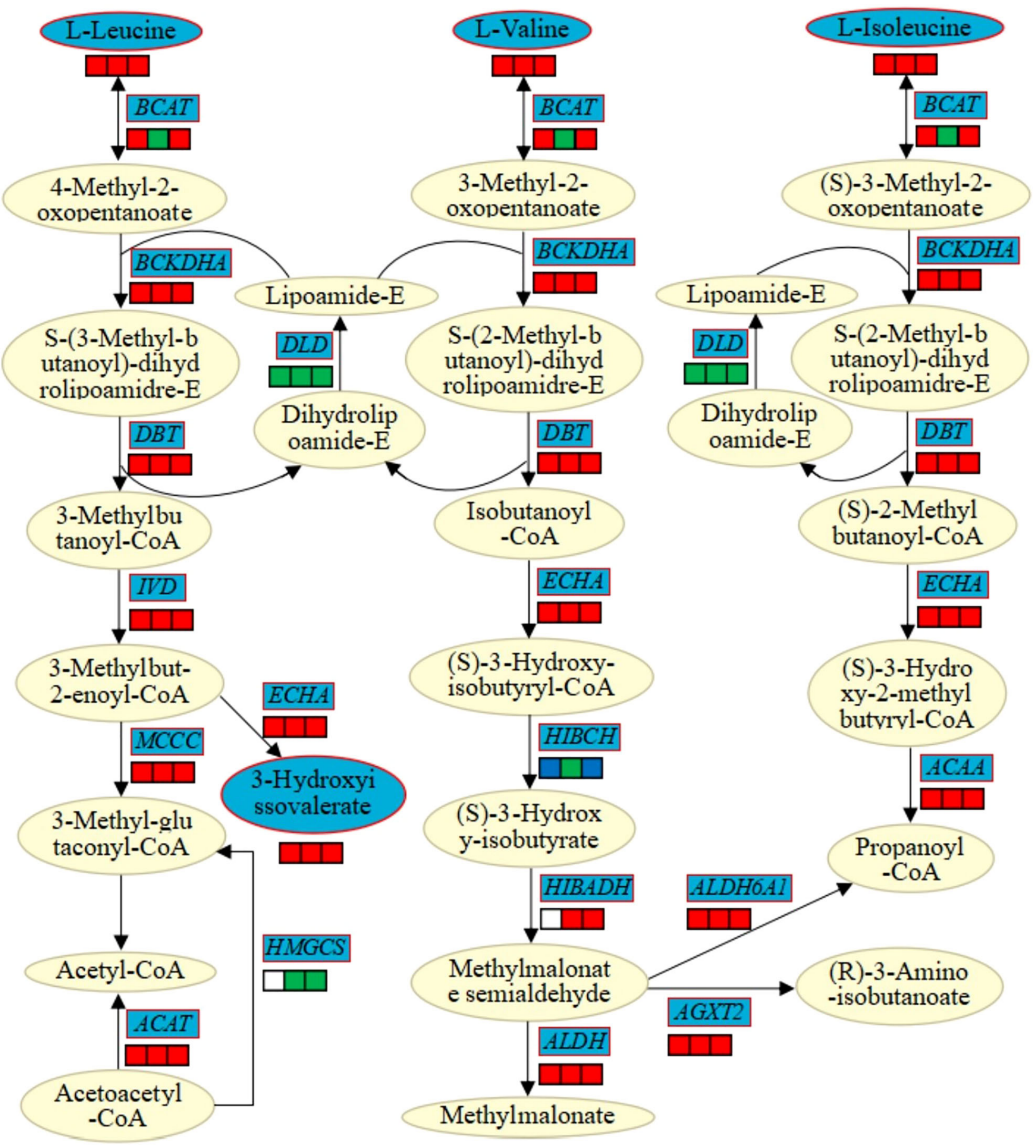
The DEGs and DAMs involved in the pathway of valine, leucine and isoleucine degradation in response to Cd stress and the addition of MT. *BCAT*, branched-chain amino acid aminotransferase; *BCKDHA*, 2-oxoisovalerate dehydrogenase E1 component alpha subunit; *DLD*, dihydrolipoamide dehydrogenase; *DBT*, 2-oxoisovalerate dehydrogenase E2 component (dihydrolipoyl transacylase); *IVD*, isovaleryl-CoA dehydrogenase; *ECHA*, enoyl-CoA hydratase; *MCCC*, 3-methylcrotonyl-CoA carboxylase alpha subunit; *ACAT*, acetyl-CoA C-acetyltransferase; *HMGCS*, hydroxymethylglutaryl-CoA synthase; *HIBCH*, 3-hydroxyisobutyryl-CoA hydrolase; *HIBADH*, 3-hydroxyisobutyrate dehydrogenase; *AGXT2*, alanine-glyoxylate transaminase/(R)-3-amino-2- methylpropionate-pyruvate transaminase; *ALDH*, aldehyde dehydrogenase (NAD+); *ALDH6A1*, malonate-semialdehyde dehydrogenase (acetylating)/methylmalonate-semialdehyde dehydrogenase; *ACAA*, acetyl-CoA acyltransferase. The blue pattern represents the DEGs or DEGs that changed under Cd stress and the addition of MT. The rectangle is divided into three equal parts (the left of rectangle represents DEGs or DEMs in CK vs T1, the middle of rectangle represents DEGs or DEMs in CK vs T2, the right of rectangle represents DEGs or DEMs in CK vs T3). The color in the rectangle represents the DEGs or DEMs are regulated under Cd stress and the addition of MT (red indicates up-regulation, green indicates down-regulation, blue indicates both up-regulation and down-regulation, white indicates neither up-regulation nor down-regulation).

The interaction of DEGs and DAMs involved in the pathway of valine, leucine and isoleucine degradation was analyzed to clarify the effects of MT on genes and metabolites related to this metabolic pathway in cotton under Cd stress (**Figure 6**). It was found that 31, 39, and 40 DEGs were associated with the pathway of valine, leucine and isoleucine degradation in CK vs T1, CK vs T2, CK vs T3, but not found in T1 vs T2, T1 vs T3, T2 vs T3. Among them, *BCKDHA* (2-oxoisovalerate dehydrogenase E1 component alpha subunit; *gene-LOC107914135*, *gene-LOC107957713*), *DBT* (2-oxoisovalerate dehydrogenase E2 component (dihydrolipoyl transacylase; *gene-LOC107931504*, *gene-LOC107935162*), *IVD* (isovaleryl-CoA dehydrogenase; *gene-LOC107889251*, *gene-LOC107891492*), *ECHA* (enoyl-CoA hydratase; *gene-LOC107952046*, *gene-LOC107910232*), *MCCC* (3-methylcrotonyl-CoA carboxylase alpha subunit; *gene-LOC107953393*, *gene-LOC107938005*, *gene-LOC107914801*, *gene-LOC107951384*), *ACAT* (acetyl-CoA C-acetyltransferase; *gene-LOC107963730*, *gene-LOC107960146*), *ALDH* (aldehyde dehydrogenase; *gene-LOC107900603*, *gene-LOC121224459*, *gene-LOC121211266*, *gene-LOC107909633*, *gene-LOC107955587*), *AGXT2* (alanine-glyoxylate transaminase/(R)-3-amino-2-methylpropionate-pyruvate transaminase; *gene-LOC107896807*, *gene-LOC107903538*, *gene-LOC107935989*, *gene-LOC121206319*), *ALDH6A1* (malonate-semialdehyde dehydrogenase (acetylating)/methylmalonate-semialdehyde dehydrogenase; *gene-LOC107886997*, *gene-LOC107908919*, *gene-LOC107908920*), *ACAA* (acetyl-CoA acyltransferase; *gene-LOC107897747*, *gene-LOC107915059*, *gene-LOC107924670*) and other DEGs were up-regulated, and *DLD* (dihydrolipoamide dehydrogenase, *gene-LOC107926533*) was down-regulated. In addition, *BCAT* (branched-chain amino acid aminotransferase; *gene-LOC107905173*, *gene-LOC107905209*, *gene-LOC107930548*, *gene-LOC107957884*, *gene-LOC121217891*) was up-regulated in CK vs T1, CK vs T2, CK vs T3, and only down-regulated in CK vs T2 that regulated by *gene-LOC107951135*. *HMGCS* (hydroxymethylglutaryl-CoA synthase, *gene-LOC107963679*) was down-regulated, and *HIBADH* (3-hydroxyisobutyrate dehydrogenase, *gene-LOC107908489*) was up-regulated in CK vs T2 and CK vs T3. *HIBCH* (3-hydroxyisobutyryl-CoA hydrolase; *novel.2291*, *gene-LOC107956866*) regulated by *gene-LOC107956866* was down-regulated in CK vs T1, CK vs T2, CK vs T3, and regulated by *novel.2291* was up-regulated in CK vs T1 and CK vs T3. HIBCH (3-hydroxyisobutyryl-CoA hydrolase; *novel.2291*, *gene-LOC107956866*) regulated by *gene LOC107956866* was down-regulated in all three combinations, and regulated by *novel.2291* was up-regulated in T1 vs T2, T2 vs T3. Therefore, it indicated that DEGs and DAMs related to the pathway of valine, leucine and isoleucine degradation were co-responded to Cd stress by adding melatonin in cotton.

### Comprehensive analysis of related DEGs and DAMs in ABC transporter pathway

The interaction of DEGs and DAMs involved in the pathway of ABC transporter was analyzed to clarify the effects of MT on genes and metabolites related to this metabolic pathway in cotton under Cd stress (**Figure S4**). A total of 51, 63, and 65 DEGs were found to be associated with the pathway of ABC transporter in CK vs T1, CK vs T2, CK vs T3, but not found in T1 vs T2 and T2 vs T3, only L-ornithine was negatively correlated with *gene-LOC107922854*. Mineral and organic ion transporters, such as glycine betaine/proline and osmoprotectant; oligosaccharide, polyol, and lipid transporters, such as nucleoside; monosaccharide transporters, such as glucose/arabinose, glucose/mannose and myo-Inositol; phosphate and amino acid transporters, such as histidine, S-methylcysteine, arginine/lysine/histidine/glutamine, arginine/lysine/histidine, branched-chain amino acid and neutral amino acid/histidine were up-regulated in CK vs T1, CK vs T2, CK vs T3. Oligogalacturonide and ribose/autoinducer2 in CK vs T3, glutamine and aspartate/glutamate/glutamine in CK vs T1, and lysine and riboflavin in CK vs T1 and CK vs T2 were up-regulated, respectively. Arginine/omithine and glutathione were down-regulated in CK vs T3. Lysine/arginine/omithine was up-regulated in CK vs T1 and CK vs T2, and down-regulated in CK vs T3. The eukaryotic ABC transporters, including ABCA Subfamily ABCA 3, ABCB Subfamily ABCB 1, ABCC Subfamily ABCC 2 were both up-regulated and down-regulated in CK vs T1, CK vs T2, CK vs T3.

## Discussion

### The relationship between melatonin and physiology response to cadmium

Heavy metal pollution in soil is one of the most important factors restricting agriculture development and endangering human health. Hyperaccumulators, such as sedum, solanum, rapeseed, mustard, clover, switchgrass, were used to adsorb or degrade heavy metal pollutants in the soil, which as an ideal method at present being the characteristics of simple, low cost, little damage to farmland, suitable for large-area treatment and not easy to cause secondary pollution (Yu et al., 2019; Nazli et al., 2020; Xu et al., 2020). However, compared with the super-accumulating bacteria, cotton is an inedible cash crop with a large biomass and a strong ability to absorb and accumulate Cd (Li et al., 2020). It is an ideal crop for remediation of Cd-contaminated soil, with unique ecological and economic advantages. This study investigated the possible mechanism of MT alleviating Cd toxicity in cotton. In this study, the growth of the cotton seedlings with MT was better than that treated with 200 *µ*mol L^−1^ Cd stress alone, but both were inferior to the control (Fig 1-A). Furthermore, the activities of SOD, POD and APX, and MDA content were increased significantly under 200 *µ*mol L^−1^ Cd stress, SOD activity first increased and then decreased significantly, the activities of POD and APX decreased significantly, while APX activity increased significantly by adding 100 *µ*mol L^−1^ MT. These results were consistent with previous study of wheat seedlings by (Ni et al., 2018) and cucumber (*Cucumis sativus* L.) plants by (Shah et al., 2020a), which indicated that the activities of APX and SOD were increased by MT to reduce Cd toxicity. The same conclusions have been also obtained from the studies on mallow (*Malva parviflora*) (Tousi et al., 2020), strawberry seedlings (Wu et al., 2021), and maize (Ma et al., 2021) plants under Cd stress, indicating that MT maybe improve Cd tolerance by activating the antioxidant defense system and reducing the oxidative stress of the plants. It is inferred that a high concentration of MT has a stronger ability to scavenge free radicals and inhibit the generation of free radicals, which in turn consumes more SOD, resulting in a relatively low SOD activity. Then H_2_O_2_ is further catalyzed by POD and APX, and or directly cleared by MT (Haider et al., 2021). In this experiment, cotton seedlings were mainly tolerant to Cd toxicity by enhancing APX activity and up-regulating genes related to ascorbic acid metabolism pathway. Therefore, the POD activity was relatively low, but the difference was not significant. Moreover, the MDA content increased significantly in this study, it decreased slightly at 100 *µ*mol L^−1^ MT, but the difference was not significant. Cadmium stress enhanced the degree of peroxidation of membrane lipids, as evidenced by the increase in MDA content (Singh et al., 2019). However, MT induced the content of MDA in wheat seedlings (Kaya et al., 2019) and cucumber plants (Shah et al., 2020a) decreased under Cd stress, which may be related to the concentration of MT. It indicated that low concentration MT was more susceptible to membrane damage than the high concentration, while high concentration has a better mitigation effect on Cd toxicity. Meanwhile, Cd concentration increased with the increase of MT concentration, especially in the roots in this experiment. This may be related to the mechanism of MT regulating the absorption, transport and accumulation of Cd. The study showed that the regulation of *IRT1*, *Nramp1*, *HMA2*, *HMA4* and *HMA3* genes by MT may promote Cd uptake and transport to the xylem, and exacerbate Cd sequestration in root vacuoles (Wang et al., 2019), and ABC transporters can assist the transport of Cd to the xylem through the apoplast to transport and fix Cd (Winter et al., 2015). The increase of alkaloids and flavonoids in plants to reduce Cd poisoning through chelation and passivation by addition of MT (Cai et al., 2017; Dhalaria et al., 2020), this is consistent with the results of this experiment on cotton plants. It was concluded that the tolerance of cotton plants to Cd and the accumulation of Cd were relatively increased due to the addition of MT. Therefore, it is believed that 100 *µ*mol L^−1^ MT has a better mitigation effect on Cd toxicity based on the analysis of the morphological and physiological responses of cotton seedlings under Cd stress by MT.

### Melatonin promotes the expression of DEGs under cadmium stress

Transcriptomics can help us understand the differences between plants at different developmental stages or different environmental conditions. These results indicate that the number of responding genes is regulated by heavy metal stress, and these DEGs may be used as functional genes to explore metabolic pathways and response mechanisms (Wang et al., 2022b). In this study, the numbers of DEGs in all treatments compared with the control were significantly higher than that between different treatments, indicating that it had a significant impact on the gene expression of cotton seedlings under Cd treatment and the addition of MT, and MT promoted the expression of certain genes under Cd stress. Meanwhile, the DEGs were significantly enriched in the pathways of photosynthesis-antenna proteins, ribosome, MAPK signaling pathway, plant-pathogen interaction, plant hormone signal transduction, porphyrin and chlorophyll metabolism, amino sugar and nucleotide sugar metabolism, starch and sucrose metabolism, galactose metabolism, valine, leucine and isoleucine degradation. In the same study, it was found that DEGs was enriched in secondary metabolites, starch and sucrose metabolism, flavonoid synthesis, phenylalanine metallization and biosynthesis in the roots of cotton seedlings, which was related to improving the activities of antioxidant system, repair system and transportation system, and reduced the toxicity of Cd (Chen et al., 2019). Moreover, 9 DEGs were all up-regulated in T2 vs T3, and involved in the pathways of ascorbic acid and aldonic acid metabolism, α-linolenic acid metabolism, MAPK signaling pathway, glycerophospholipid metabolism and plant hormone signal transduction. The expression of 9 DEGs maybe preliminarily explain why the detoxification effect on Cd by 100 *µ*mol L^−1^ MT was better than that of 50 *µ*mol L^−1^ MT. In particular, ascorbic acid had strong reducing properties and was present in mitochondria, chloroplasts, peroxisomes, nuclei, cytoplasm, endoplasmic reticulum and vacuoles (Dumanović et al., 2021). Cadmium toxicity in tomato (Elkelish et al., 2020) and cucumber (Semida et al., 2018) seedlings can be reduced by exogenous ascorbic acid. This was due to ascorbic acid acted as a special electron donor for APX to reduce the content of H_2_O_2_ and O^2−^, and was oxidized to dehydroascorbic acid (DHAA) and then non-enzymatically reduced by GSH to form an AsA-GSH cycle, which eliminates reactive oxygen species (ROS) produced under heavy metal stress by redox regulation (Intarasit & Saengnil, 2021). Studies have found that Cd toxicity reprogrammed the gene transcription profile of the AsA-GSH cycle, the expression level of the corresponding DEGs was greater especially in Cd-tolerant wheat varieties (Zhang et al., 2021b), and the addition of exogenous MT increased the content of ascorbic acid and GSH in safflower seedlings (Amjadi et al., 2021), which was consistent with the up-regulated of genes related to the ascorbic acid metabolism pathway in this study, and the pretreatment with 2-OHMT (Shah et al., 2020b) and MT (Shah et al., 2020c) increased the accumulation of glutathione in *Cucumis sativus* seedlings, indicating that plant seedlings can improve the tolerance to Cd toxicity by accelerating the AsA-GSH cycle.

### Melatonin promotes metabolism of alkaloids and flavonoids under cadmium stress

Metabolites, as the end products of cell activities, are a direct reflection of the effects of temporal and spatial changes or environmental changes on plant cells. The UPLC-MS/MS was used to conduct qualitative and quantitative analysis of widely targeted metabolites in cotton seedling leaf samples affected by Cd stress and MT addition. In our study, the number of up-regulated DAMs was significantly higher than that of down-regulated in all treatments compared with the control. Isoquinoline, α-solasonine, etc. had higher up-regulation multiples, and most of them were alkaloids; N-acetyl-L-glutamic acid, as one of amino acids and their derivatives, had higher down-regulation multiples. These findings indicated MT treatment caused the synthesis of isoquinolines, indoles and other alkaloids in cotton leaves, and inhibited or reduced the synthesis of N-Acetyl-L-glutamic acid to a certain extent, which may be related to the tolerance to Cd in plant. Studies have shown that Cd increased the content of alkaloid and changed its composition of *Narcissus tazetta* plants (Soleimani et al., 2020), increased the production of indole alkaloids (vindoline, vinblastine and vinblastine) in *Catharanthus roseus* seedlings, and the gene expression of enzymes and alkaloid transporters in TIA pathway in leaves (Chen et al., 2017). These studies were consistent with our findings, which respond to Cd stress by increasing the accumulation of alkaloids in plants. The functional groups of elements such as amino, carboxyl, and hydroxyl in amino acids or lipids can determine the chelating agents in plant vacuoles such as chelating agents, metallothionein, etc., followed by limited vacuoles, cell walls, etc., to achieve passivation and heavy metal detoxification (Dhalaria et al., 2020). Furthermore, tyrosine was the starting material for the synthesis of isoquinoline alkaloids, so the synthesis of isoquinoline alkaloids also needs to consume a certain amount of tyrosine (Liu et al., 2022). Therefore, it caused the down-regulation of amino acids and their derivatives. Our study also found that the number of up-regulated DAMs decreased significantly under Cd stress by adding MT, and a large number of down-regulated DAMs was caused by 100 *µ*mol L^−1^ MT. The flavonoids, such as chrysin-5-O-glucoside, genistein-7-O-(6’’-malonyl) glucoside, etc. had higher up-regulation multiples, it was consistent with the study of *Robinia pseudoacacia* seedlings (Zhang et al., 2021a) and *Cucumis sativus* seedlings (Shah et al., 2020b) respond to Cd stress by increasing the total flavonoid content. It was speculated that the oxidative activity of ROS can be reduced by flavonoids in plants grown in Cd-contaminated soil, due to its antioxidant properties and the capability of chelating Cd by hydroxyl or carboxyl groups, thereby reducing the toxicity of heavy metal (Khalid et al., 2019). Therefore, MT treatment caused a significant up-regulation of flavonoid metabolites, which would help improve the resistance to Cd stress. Moreover, 9,12,13-Trihydroxy-10,15-octadecadienoic acid and LysoPC series, had higher down-regulation multiples, were all lipids. It was speculated that the down-regulated DEGs maybe inhibit or reduce the expression of some DAMs, especially the down-regulation of amino acids and their derivatives, such as L-slanyl-L-phenylalanine, L-valyl-L-leucine, L-valyl-L-phenylalanine, L-leucyl-L-leucine etc. caused by 100 *µ*mol L^−1^ MT, or maybe the combination of lipids or amino acids with chelating agents, so as to reduce the toxicity of Cd to plants (Cai et al., 2017).

### Co-enrichment of DEGs and DEMs enhances the tolerance to cadmium

A great deal of information about the metabolic pathway (KEGG) were obtained by integrating the transcriptomic data with metabolomic data. The metabolic pathway of valine, leucine and isoleucine degradation was one of the most significant ways to enrich DEGs and DAMs under Cd sress after MT addition. Leucine, isoleucine and valine are all branched chain amino acids, which can promote the secretion of growth hormone to promote the normal growth of plants, repair damaged tissues, and quickly decompose and convert into glucose to provide energy. The study on tomato had found that Cd treatment caused an up-regulation of leucine aminopeptidase-A and increased the hydrolysis activities of Leu, Met, Arg, Pro and Lys in roots (Boulila-Zoghlami et al., 2011). It was inferred that the decomposition of leucine, isoleucine and valine under Cd stress after MT addition was accelerated by DEGs and DAMs significantly enriched in this pathway, and provided energy to maintain plant growth and respond to Cd stress. A total of 33 DEGs and 4 DAMs were involved in this metabolic pathway, 30 DEGs were positively correlated to promote the metabolism, and only *gene-LOC107926533*, *gene-LOC107956866* and *gene-LOC107963679* were negatively correlated to inhibit or slow down the metabolism. Meanwhile, β-hydroxyisovaleric acid, as a metabolite of leucine, had functions such as improving the recovery ability of tissue damage and improving basic metabolism, which was related to the function of this metabolic pathway respond to Cd stress. Our study also found that ABC transporter metabolic pathway was another pathway that DEGs and DAMs were significantly enriched under Cd stress after MT addition. A total of 46 DEGs and 16 DAMs were involved, and 11 amino acids were riched in this metabolic pathway and participated in the regulation of amino acid metabolism. Studies have found that a large number of ABC transporter genes were induced or inhibited under Cd stress (Chen et al., 2019), and AtABCC1 and AtABCC2, as ABCC-type transporter, were determined as the main apo-phytochelatin and phytochelatin-heavy metal (oid) complex transporters (Klein et al., 2006), so it was very important for the detoxification of Cd. However, some studies have also found that ABC transporter can assist the transport of Cd to the xylem through the ectoplasmic pathway to transport and fix Cd (Winter et al., 2015). Although ABC transporter was involved in the tolerance and detoxification of heavy metals in plants, its mechanism needs to be further explored (Klein et al., 2006).

## Conclusion

In the present study, the combined analysis of transcriptomic and metabolomic revealed the complex response mechanisms induced by Cd stress, and clarified the metabolic pathways by which MT enhanced the tolerance of cotton seedlings to Cd stress. The growth of cotton seedlings was affected by Cd stress, while the activities of APX and SOD increased by adding MT to reduce the Cd toxicity to plants. The DEGs were significantly affected by Cd stress and the addition of melatonin, especially only 9 DEGs were found between different concentrations of melatonin, which all were up-regulated. The synthesis of alkaloids and flavonoids were promoted, and the synthesis of lipids, amino acids and their derivatives were inhibited or reduced by melatonin under Cd stress. The co-expression of DEGs and DAMs related to the pathways of valine, leucine and isoleucine degradation, ABC transporter, alpha-linolenic acid metabolism can be regulated by adding MT under Cd stress, and the toxicity of Cd to cotton seedlings can be relieved by MT.

## Data availability statement

The datasets presented in this study can be found in online repositories. The names of the repository/repositories and accession number(s) can be found in the article/**Supplementary Material**.

## Author contributions

XKW, LL, XY, JL, XW assisted in doing experiments and measuring indicators, analyzed the data and provided suggestions. All authors contributed to the article and approved the submitted version.

## Funding

The work supported by the funding from the National Natural Science Foundation of China (31960414, 32260454), High-level Talent Fund of Scientific Research for Intrduction and Training in Yan’an, Shaanxi Province of China (2019-06), Research Project of Yan’an University (YDZ2019-07), Industry-University-Research Project of Yan’an University (CXY202112) and the research project of soil remediation and utilization of construction waste landfill.

## Acknowledgments

We thank the anonymous reviewers for their helpful comments.

## Conflict of interest

The authors declare that the research was conducted in the absence of any commercial or financial relationships that could be construed as a potential conflict of interest.

## Publisher’s note

All claims expressed in this article are solely those of the authors and do not necessarily represent those of their affiliated organizations, or those of the publisher, the editors and the reviewers. Any product that may be evaluated in this article, or claim that may be made by its manufacturer, is not guaranteed or endorsed by the publisher.

## References

[B1] Amjadi Z. Namdjoyan S. Abolhasani Soorki A. (2021). Exogenous melatonin and salicylic acid alleviates cadmium toxicity in safflower (Carthamus tinctorius l.) seedlings. Ecotoxicology 30, 387–401. doi: 10.1007/s10646-021-02364-y 33624206

[B2] Arbona V. Manzi M. Ollas C. D. Gómez-Cadenas A. (2013). Metabolomics as a tool to investigate abiotic stress tolerance in plants. Int. J. Mol. Sci. 14, 4885–4911. doi: 10.3390/ijms14034885. 23455464 PMC3634444

[B3] Arnao M. B. Hernández-Ruiz J. (2015). Functions of melatonin in plants: a review. J. Pineal Res. 59, 133–150. doi: 10.1111/jpi.12253 26094813

[B4] Beyer W. F. Fridovich I. (1987). Assaying for superoxide dismutase activity: Some large consequences of minor changes in conditions. Anal. Biochem. 161, 559–566. doi: 10.1016/0003-2697(87)90489-1 3034103

[B5] Boulila-Zoghlami L. Gallusci P. Holzer F. M. Basset G. J. Djebali W. Chaïbi W.. (2011). Up-regulation of leucine aminopeptidase-a in cadmium-treated tomato roots. Planta 234, 857. doi: 10.1007/s00425-011-1468-y 21744092

[B6] Byeon Y. Lee H. Y. Hwang O. J. Lee H. J. Lee K. Back K. (2015). Coordinated regulation of melatonin synthesis and degradation genes in rice leaves in response to cadmium treatment. J. pineal Res. 58, 470–478. doi: 10.1111/jpi.12232 25783167

[B7] Cai S.-Y. Zhang Y. Xu Y.-P. Qi Z.-Y. Li M.-Q. Ahammed G. J.. (2017). HsfA1a upregulates melatonin biosynthesis to confer cadmium tolerance in tomato plants. J. Pineal Res. 62, e12387. doi: 10.1111/jpi.12387 28095626

[B8] Chen G.-X. Asada K. (1989). Ascorbate peroxidase in tea leaves: occurrence of two isozymes and the differences in their enzymatic and molecular properties. Plant Cell Physiol. 30, 987–998. doi: 10.1093/oxfordjournals.pcp.a077844

[B10] Chen P. Chen T. Li Z. Jia R. Luo D. Tang M.. (2020). Transcriptome analysis revealed key genes and pathways related to cadmium-stress tolerance in kenaf (Hibiscus cannabinus l.). Ind. Crops Prod. 158, 112970. doi: 10.1016/j.indcrop.2020.112970

[B9] Chen H. Li Y. Ma X. Guo L. He Y. Ren Z.. (2019). Analysis of potential strategies for cadmium stress tolerance revealed by transcriptome analysis of upland cotton. Sci. Rep. 9, 86. doi: 10.1038/s41598-018-36228-z 30643161 PMC6331580

[B11] Chen Q. Wu K. Tang Z. Guo Q. Guo X. Wang H. (2017). Exogenous ethylene enhanced the cadmium resistance and changed the alkaloid biosynthesis in catharanthus roseus seedlings. Acta Physiol. Plant. 39, 267. doi: 10.1007/s11738-017-2567-6

[B12] Chen S. Zhou Y. Chen Y. Gu J. (2018). Fastp: an ultra-fast all-in-one FASTQ preprocessor. Bioinformatics 34, i884–i890. doi: 10.1093/bioinformatics/bty560 30423086 PMC6129281

[B13] Dhalaria R. Kumar D. Kumar H. Nepovimova E. Kuča K. Torequl Islam M.. (2020). Arbuscular mycorrhizal fungi as potential agents in ameliorating heavy metal stress in plants. Agronomy 10, 815. doi: 10.3390/agronomy10060815

[B14] Dumanović J. Nepovimova E. Natić M. Kuča K. Jaćević V. (2021). The significance of reactive oxygen species and antioxidant defense system in plants: A concise overview. Front. Plant Sci. 11. doi: 10.3389/fpls.2020.552969 PMC7815643 33488637

[B15] Elkelish A. Qari S. H. Mazrou Y. S. A. Abdelaal K. A. A. Hafez Y. M. Abu-Elsaoud A. M.. (2020). Exogenous ascorbic acid induced chilling tolerance in tomato plants through modulating metabolism, osmolytes, antioxidants, and transcriptional regulation of catalase and heat shock proteins. Plants 9, 431. doi: 10.3390/plants9040431 PMC7238171 32244604

[B16] Gao J. Sun L. Yang X. Liu J.-X. (2013). Transcriptomic analysis of cadmium stress response in the heavy metal hyperaccumulator sedum alfredii hance. PloS One 8, e64643. doi: 10.1371/journal.pone.0064643 23755133 PMC3670878

[B17] Guo H. Guo H. Zhang L. Tang Z. Yu X. Wu J.. (2019). Metabolome and transcriptome association analysis reveals dynamic regulation of purine metabolism and flavonoid synthesis in transdifferentiation during somatic embryogenesis in cotton. Int. J. Mol. Sci. 20, 2070. doi: 10.3390/ijms20092070. PMC6539419 31027387

[B18] Haider F. U. Cai L. Q. Coulter J. A. Cheema S. A. Wu J. Zhang R. Z.. (2021). Cadmium toxicity in plants: Impacts and remediation strategies. Ecotoxicol. Environ. Saf. 211, 111887. doi: 10.1016/j.ecoenv.2020.111887 33450535

[B19] Hardeland R. (2015). Melatonin in plants and other phototrophs: Advances and gaps concerning the diversity of functions. J. Exp. Bot. 66, 627–646. doi: 10.1093/jxb/eru386 25240067

[B20] Heath R. L. Packer L. (1968). Photoperoxidation in isolated chloroplasts: I. kinetics and stoichiometry of fatty acid peroxidation. Arch. Biochem. Biophys. 125, 189–198. doi: 10.1016/0003-9861(68)90654-1 5655425

[B21] Huang Y. Li D. Zhao L. Chen A. Li J. Tang H.. (2019). Comparative transcriptome combined with physiological analyses revealed key factors for differential cadmium tolerance in two contrasting hemp (Cannabis sativa l.) cultivars. Ind. Crops Prod. 140, 111638. doi: 10.1016/j.indcrop.2019.111638

[B22] Hussain A. Mun B.-G. Imran Q. M. Lee S.-U. Adamu T. A. Shahid M.. (2016). Nitric oxide mediated transcriptome profiling reveals activation of multiple regulatory pathways in arabidopsis thaliana. Front. Plant Sci. 7. doi: 10.3389/fpls.2016.00975 PMC4926318 27446194

[B23] Intarasit S. Saengnil K. (2021). Transient production of H2O2 and NO induced by ascorbic acid coincides with promotion of antioxidant enzyme activity and reduction of pericarp browning of harvested longan fruit. Sci. Hortic. 277, 109784. doi: 10.1016/j.scienta.2020.109784

[B25] Jiang N. Fan X. Lin W. Wang G. Cai K. (2019). Transcriptome analysis reveals new insights into the bacterial wilt resistance mechanism mediated by silicon in tomato. Int. J. Mol. Sci. 20, 761. doi: 10.3390/ijms20030761 PMC6387441 30754671

[B24] Jiang J. Ren X. Li L. Hou R. Sun W. Jiao C.. (2020). H2S regulation of metabolism in cucumber in response to salt-stress through transcriptome and proteome analysis. Front. Plant Sci. 11. doi: 10.3389/fpls.2020.01283 PMC7466724 32973842

[B26] Kaya C. Okant M. Ugurlar F. Alyemeni M. N. Ashraf M. Ahmad P. (2019). Melatonin-mediated nitric oxide improves tolerance to cadmium toxicity by reducing oxidative stress in wheat plants. Chemosphere 225, 627–638. doi: 10.1016/j.chemosphere.2019.03.026 30901656

[B27] Khalid M. Saeed ur R. Bilal M. Huang D.-f. (2019). Role of flavonoids in plant interactions with the environment and against human pathogens — a review. J. Integr. Agric. 18, 211–230. doi: 10.1016/S2095-3119(19)62555-4

[B28] Kim D. Langmead B. Salzberg S. L. (2015). HISAT: a fast spliced aligner with low memory requirements. Nat. Methods 12, 357–360. doi: 10.1038/nmeth.3317 25751142 PMC4655817

[B29] Klein M. Burla B. Martinoia E. (2006). The multidrug resistance-associated protein (MRP/ABCC) subfamily of ATP-binding cassette transporters in plants. FEBS Lett. 580, 1112–1122. doi: 10.1016/j.febslet.2005.11.056 16375897

[B34] Liao Y. Smyth G. K. Shi W. (2014). featureCounts: an efficient general purpose program for assigning sequence reads to genomic features. Bioinformatics 30, 923–930. doi: 10.1093/bioinformatics/btt656. 24227677

[B32] Li H. Chang J. J. Chen H. J. Wang Z. Y. Gu X. R. Wei C. H.. (2017). Exogenous melatonin confers salt stress tolerance to watermelon by improving photosynthesis and redox homeostasis. Front. Plant Sci. 1. doi: 10.3389/fpls.2017.00295 PMC5331065 28298921

[B31] Li D. Gaquerel E. (2021). Next-generation mass spectrometry metabolomics revives the functional analysis of plant metabolic diversity. Annu. Rev. Plant Biol. 72, 867–891. doi: 10.1146/annurev-arplant-071720-114836 33781077

[B36] Liu N. Li J. Lv J. Yu J. Xie J. Wu Y.. (2021b). Melatonin alleviates imidacloprid phytotoxicity to cucumber (Cucumis sativus l.) through modulating redox homeostasis in plants and promoting its metabolism by enhancing glutathione dependent detoxification. Ecotoxicol. Environ. Saf. 217, 112248. doi: 10.1016/j.ecoenv.2021.112248 33901782

[B35] Liu C. M-m L. E-k He Yao A.-j. Wang G.-b. Tang Y.-t.. (2021a). Phenomic and metabolomic responses of roots to cadmium reveal contrasting resistance strategies in two rice cultivars (Oryza sativa l.). Soil Ecol. Lett. 3, 220–229. doi: 10.1007/s42832-021-0088-0

[B37] Liu X.-M. Tan J.-P. Cheng S.-Y. Chen Z.-X. Ye J.-B. Zheng J.-R.. (2022). Comparative transcriptome analysis provides novel insights into the molecular mechanism of berberine biosynthesis in coptis chinensis. Sci. Hortic. 291, 110585. doi: 10.1016/j.scienta.2021.110585

[B33] Li L. Yan X. Li J. Tian Y. (2021). Physiological and FtCHS gene expression responses to PEG-simulated drought and cadmium stresses in tartary buckwheat seedlings. J. Plant Growth Regul. doi: 10.1007/s00344-021-10530-z

[B30] Li C. Zheng C. Zhou K. Han W. Tian C. Ye S.. (2020). Toleration and accumulation of cotton to heavy metal - potential use for phytoremediation. Soil Sediment Contam.: Int. J. 29, 516–531. doi: 10.1080/15320383.2020.1747979

[B38] Love M. I. Huber W. Anders S. (2014). Moderated estimation of fold change and dispersion for RNA-seq data with DESeq2. Genome Biol. 15, 550. doi: 10.1186/s13059-014-0550-8 25516281 PMC4302049

[B39] Ma L. Huang Z. Li S. Ashraf U. Yang W. Liu H.. (2021). Melatonin and nitrogen applications modulate early growth and related physio-biochemical attributes in maize under cd stress. J. Soil Sci. Plant Nutr. 21, 978–990. doi: 10.1007/s42729-021-00415-1

[B40] Navarro-Reig M. Jaumot J. Piña B. Moyano E. Galceran M. T. Tauler R. (2017). Metabolomic analysis of the effects of cadmium and copper treatment in oryza sativa l. using untargeted liquid chromatography coupled to high resolution mass spectrometry and all-ion fragmentation. Metallomics 9, 660–675. doi: 10.1039/c6mt00279j. 28480907

[B41] Nazli F. Mustafa A. Ahmad M. Hussain A. Jamil M. Wang X.. (2020). A review on practical application and potentials of phytohormone-producing plant growth-promoting rhizobacteria for inducing heavy metal tolerance in crops. Sustainability 12, 9056. doi: 10.3390/su12219056.

[B42] Ni J. Wang Q. Shah F. A. Liu W. Wang D. Huang S.. (2018). Exogenous melatonin confers cadmium tolerance by counterbalancing the hydrogen peroxide homeostasis in wheat seedlings. Molecules 23, 799. doi: 10.3390/molecules23040799 PMC6017192 29601513

[B43] Pütter J. (1974). Peroxidases: Methods of enzymatic analysis (Second edition). Ed. Bergmeyer H. U. (Verlag Chemie Weinheim: Academic Press New York and London), 685–690.

[B44] Qin G. Niu Z. Yu J. Li Z. Ma J. Xiang P. (2021). Soil heavy metal pollution and food safety in China: Effects, sources and removing technology. Chemosphere 267, 129205. doi: 10.1016/j.chemosphere.2020.129205 33338709

[B45] Rezzani R. Franco C. Hardeland R. Rodella L. F. (2020). Thymus-pineal gland axis: Revisiting its role in human life and ageing. Int. J. Mol. Sci. 21, 8806. doi: 10.3390/ijms21228806 PMC7699871 33233845

[B46] Semida W. M. Hemida K. A. Rady M. M. (2018). Sequenced ascorbate-proline-glutathione seed treatment elevates cadmium tolerance in cucumber transplants. Ecotoxicol. Environ. Saf. 154, 171–179. doi: 10.1016/j.ecoenv.2018.02.036 29471279

[B47] Shah A. A. Ahmed S. Ali A. Yasin N. A. (2020a). 2-hydroxymelatonin mitigates cadmium stress in cucumis sativus seedlings: Modulation of antioxidant enzymes and polyamines. Chemosphere 243, 125308. doi: 10.1016/j.chemosphere.2019.125308 31722261

[B48] Shah A. A. Ahmed S. Yasin N. A. (2020b). 2-hydroxymelatonin induced nutritional orchestration in cucumis sativus under cadmium toxicity: modulation of non-enzymatic antioxidants and gene expression. Int. J. Phytoremediation 22, 497–507. doi: 10.1080/15226514.2019.1683715 31703532

[B49] Shah A. A. Ahmed S. Yasin N. A. (2020c). Cadmium stress consolation in melatonin supplemented cucumis sativus through modulation of antioxidative defense system. Iran. J. Plant Physiol. 10, 3135–3154. doi: 10.22034/IJPP.2020.672573

[B50] Singh S. Singh V. P. Prasad S. M. Sharma S. Ramawat N. Dubey N. K.. (2019). Interactive effect of silicon (Si) and salicylic acid (SA) in maize seedlings and their mechanisms of cadmium (Cd) toxicity alleviation. J. Plant Growth Regul. 38, 1587–1597. doi: 10.1007/s00344-019-09958-1

[B51] Soleimani S. H. Bernard F. Amini M. Khavari- nezhad R.-A. (2020). Cadmium accumulation and alkaloid production of narcissus tazetta plants grown under *in vitro* condition with cadmium stress. Plant Physiol. Rep. 25, 51–57. doi: 10.1007/s40502-019-00476-6

[B52] Su L. Xie Y. He Z. Zhang J. Tang Y. Zhou X. (2021). Network response of two cherry tomato (Lycopersicon esculentum) cultivars to cadmium stress as revealed by transcriptome analysis. Ecotoxicol. Environ. Saf. 222, 112473. doi: 10.1016/j.ecoenv.2021.112473 34224970

[B53] Tousi S. Zoufan P. Ghahfarrokhie A. R. (2020). Alleviation of cadmium-induced phytotoxicity and growth improvement by exogenous melatonin pretreatment in mallow (Malva parviflora) plants. Ecotoxicol. Environ. Saf. 206, 111403. doi: 10.1016/j.ecoenv.2020.111403 33011513

[B54] Wang J. Chen X. Chu S. You Y. Chi Y. Wang R.. (2022a). Comparative cytology combined with transcriptomic and metabolomic analyses of solanum nigrum l. in response to cd toxicity. J. Hazard. Mater. 423, 127168. doi: 10.1016/j.jhazmat.2021.127168 34534808

[B56] Wang J. Duan X. Wang Y. Sheng J. (2022b). Transcriptomic and physiological analyses of miscanthus lutarioriparius in response to plumbum stress. Ind. Crops Prod. 176, 114305. doi: 10.1016/j.indcrop.2021.114305

[B55] Wang M. Duan S. H. Zhou Z. C. Chen S. H. Wang D. (2019). Foliar spraying of melatonin confers cadmium tolerance in *Nicotiana tabacum* l. Ecotoxicol. Environ. Saf. 170, 68–76. doi: 10.1016/j.ecoenv.2018.11.127 30529622

[B57] Wang J. Zhang Y. Xu N. Zhang H. Fan Y. Rui C.. (2021). Genome-wide identification of CK gene family suggests functional expression pattern against Cd2+ stress in gossypium hirsutum l. Int. J. Biol. Macromol. 188, 272–282. doi: 10.1016/j.ijbiomac.2021.07.190 34364943

[B58] Winter G. Todd C. D. Trovato M. Forlani G. Funck D. (2015). Physiological implications of arginine metabolism in plants. Front. Plant Sci. 6. doi: 10.3389/fpls.2015.00534 PMC4520006 26284079

[B59] Wu F.-B. Chen F. Wei K. Zhang G.-P. (2004). Effect of cadmium on free amino acid, glutathione and ascorbic acid concentrations in two barley genotypes (Hordeum vulgare l.) differing in cadmium tolerance. Chemosphere 57, 447–454. doi: 10.1016/j.chemosphere.2004.06.042 15350406

[B60] Wu S. Wang Y. Zhang J. Gong X. Zhang Z. Sun J.. (2021). Exogenous melatonin improves physiological characteristics and promotes growth of strawberry seedlings under cadmium stress. Hortic. Plant J. 7, 13–22. doi: 10.1016/j.hpj.2020.06.002

[B61] Xie M. Chen W. Lai X. Dai H. Sun H. Zhou X.. (2019). Metabolic responses and their correlations with phytochelatins in amaranthus hypochondriacus under cadmium stress. Environ. pollut. 252, 1791–1800. doi: 10.1016/j.envpol.2019.06.103 31299508

[B62] Xu Z. Wang D. Tang W. Wang L. Li Q. Lu Z.. (2020). Phytoremediation of cadmium-polluted soil assisted by d-gluconate-enhanced enterobacter cloacae colonization in the solanum nigrum l. rhizosphere. Sci. Total Environ. 732, 139265. doi: 10.1016/j.scitotenv.2020.139265 32416401

[B63] Yadav B. S. Singh S. Srivastava S. Mani A. (2019). Analysis of chickpea gene co-expression networks and pathways during heavy metal stress. J. Biosci. 44, 99. doi: 10.1007/s12038-019-9899-x 31502577

[B65] Yuvaraj A. Thangaraj R. Ravindran B. Chang S. W. Karmegam N. (2021). Centrality of cattle solid wastes in vermicomposting technology – a cleaner resource recovery and biowaste recycling option for agricultural and environmental sustainability. Environ. pollut. 268, 115688. doi: 10.1016/j.envpol.2020.115688 33039975

[B64] Yu H. Zou W. Chen J. Chen H. Yu Z. Huang J.. (2019). Biochar amendment improves crop production in problem soils: A review. J. Environ. Manage. 232, 8–21. doi: 10.1016/j.jenvman.2018.10.117 30466010

[B66] Zeng T. Fang B. Huang F. Dai L. Tang Z. Tian J.. (2021). Mass spectrometry-based metabolomics investigation on two different indica rice grains (Oryza sativa l.) under cadmium stress. Food Chem. 343, 128472. doi: 10.1016/j.foodchem.2020.128472 33139121

[B67] Zhang C. Jia X. Zhao Y. Wang L. Cao K. Zhang N.. (2021a). The combined effects of elevated atmospheric CO2 and cadmium exposure on flavonoids in the leaves of robinia pseudoacacia l. seedlings. Ecotoxicol. Environ. Saf. 210, 111878. doi: 10.1016/j.ecoenv.2020.111878 33418159

[B68] Zhang T. Xiao J. Zhao Y. Zhang Y. Jie Y. Shen D.. (2021b). Comparative physiological and transcriptomic analyses reveal ascorbate and glutathione coregulation of cadmium toxicity resistance in wheat genotypes. BMC Plant Biol. 21, 459. doi: 10.1186/s12870-021-03225-w 34625028 PMC8501743

[B69] Zhang X. Xu L. Zhang H. Jiang Z. Cai W. (2021c). Emergy based intelligent decision-making model for remanufacturing process scheme integrating economic and environmental factors. J. Cleaner Prod. 291, 125247. doi: 10.1016/j.jclepro.2020.125247

[B70] Zhao C. Nawaz G. Cao Q. Xu T. (2022). Melatonin is a potential target for improving horticultural crop resistance to abiotic stress. Sci. Hortic. 291, 110560. doi: 10.1016/j.scienta.2021.110560

[B71] Zhu H. Ai H. Cao L. Sui R. Ye H. Du D.. (2018). Transcriptome analysis providing novel insights for cd-resistant tall fescue responses to cd stress. Ecotoxicol. Environ. Saf. 160, 349–356. doi: 10.1016/j.ecoenv.2018.05.066 29860131

[B72] Zhu Y. Wang H. Lv X. Zhang Y. Wang W. (2020). Effects of biochar and biofertilizer on cadmium-contaminated cotton growth and the antioxidative defense system. Sci. Rep. 10, 20112. doi: 10.1038/s41598-020-77142-7 33208871 PMC7674410

