# Supplementary material for: Metabolome and transcriptome association analysis revealed key factors involved in melatonin mediated cadmium-stress tolerance in cotton
Source: Front Plant Sci. 2022 Sep 20;13:995205. doi: 10.3389/fpls.2022.995205 (PMC9530903; doi:10.3389/fpls.2022.995205)
Supplement: Supplementary file 1 [file DataSheet_1.docx]

**Manuscript title:**

Metabolome and transcriptome association analysis revealed key factors involved in melatonin mediated cadmium-stress tolerance in cotton

**Manuscript type:**

Article

**Authors:**

Ling Li, Xuyu Yan, Juan Li, Xiang Wu, Xiukang Wang^*^

**Author affiliations:**

Shaanxi Key Laboratory of Chinese Jujube, College of Life Sciences, Yan'an University, Yan'an, Shaanxi 716000, China

**Corresponding author:**

College of Life Sciences, Yan'an University, Yan'an, Shaanxi 716000, China

[wangxiukang@126.com](mailto:wangxiukang@126.com) (Xiukang Wang)

Telephone and fax: +86 9112332020

**Fig S1.** Phenotypic and physiological responses of cotton seedlings leave under Cd stress and adding melatonin.

**A**

**CK**

**T1**

**T2**

**T3**


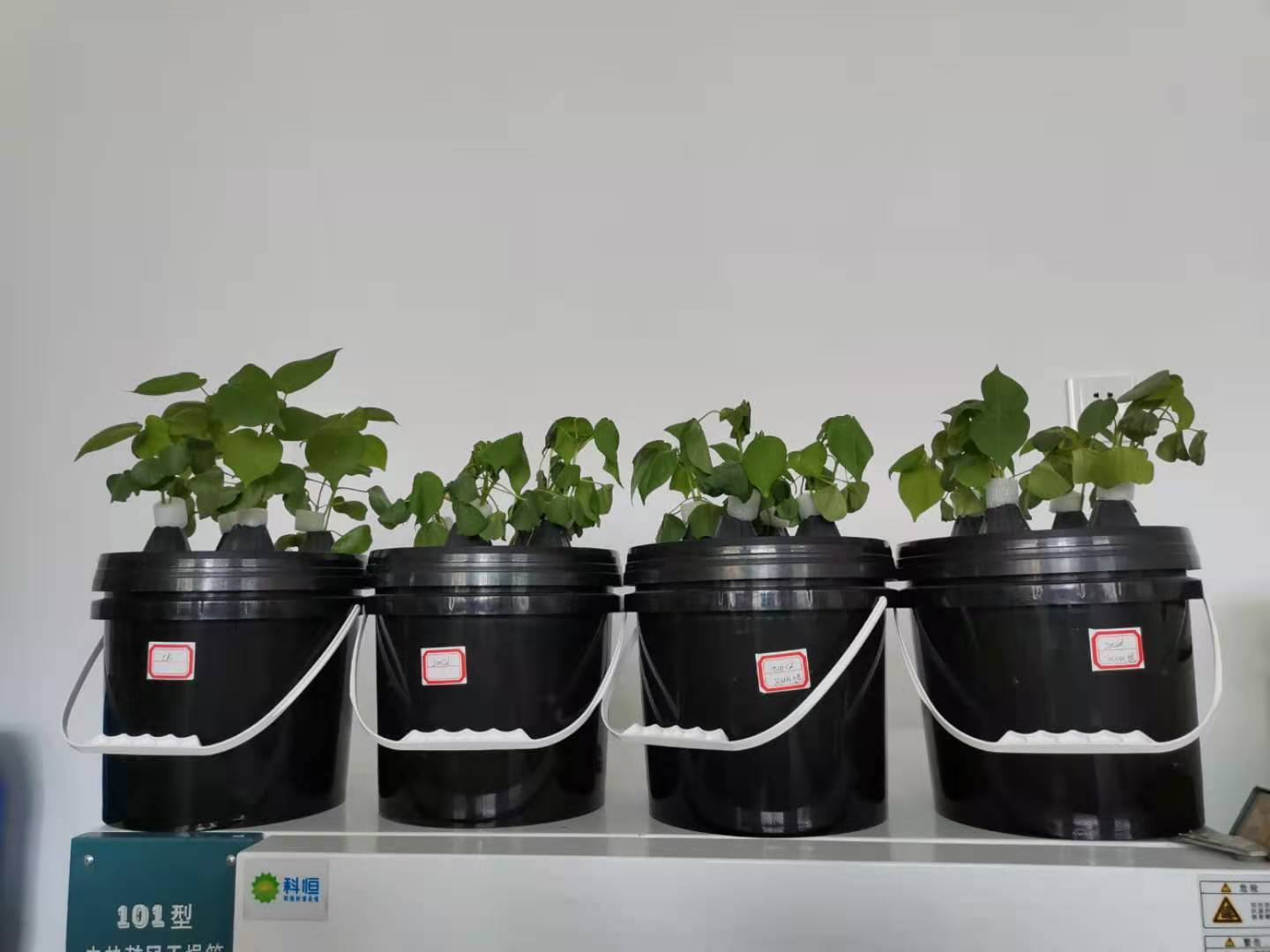

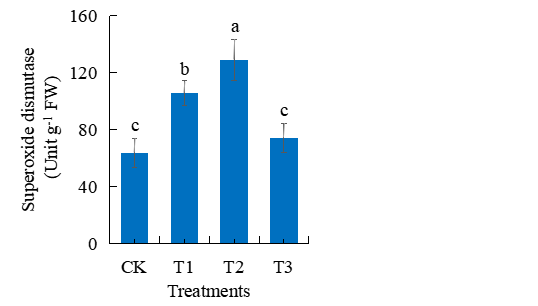

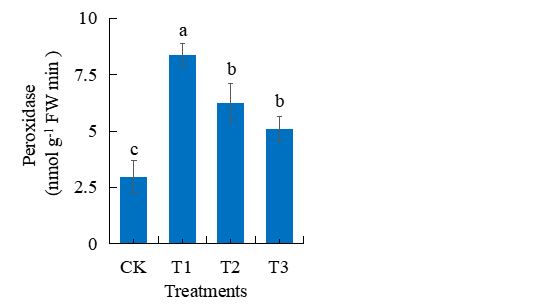


**B**


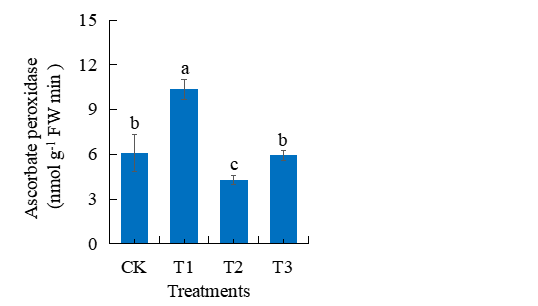

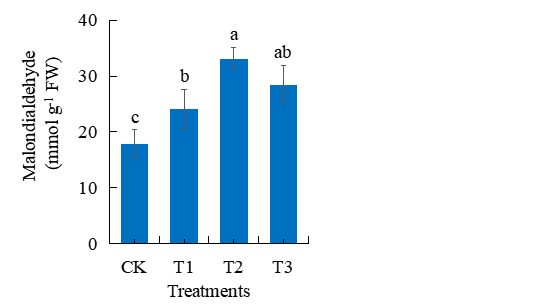


Note: CK, the control treatment; T1, 200 µmol L^−1^ Cd treatment; T2, the treatment of 50 µmol L^−1^ MT+200 µmol L^−1^ Cd; T3, the treatment of 100 µmol L^−1^ MT+200 µmol L^−1^ Cd. A, Phenotype of cotton seedlings leaves under different treatments; B, Changes in the activities of SOD, POD and APX, and the content of MDA in the cotton leaves under different treatments.

**Fig S2.** Cd concentration in cotton shoots and roots under Cd stress and adding melatonin.


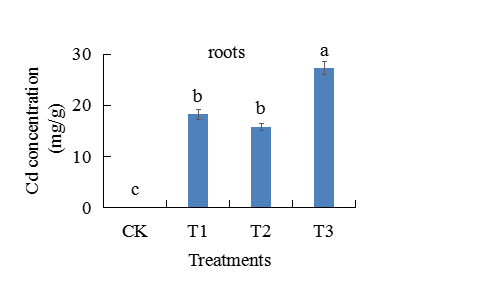

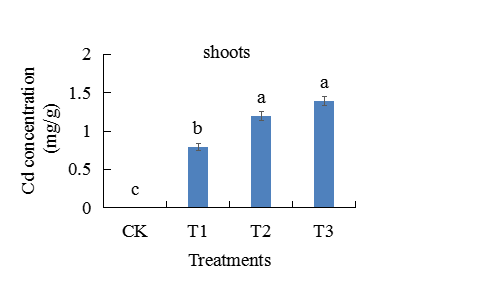


Note: CK, the control treatment; T1, 200 µmol L^−1^ Cd treatment; T2, the treatment of 50 µmol L^−1^ MT+200 µmol L^−1^ Cd; T3, the treatment of 100 µmol L^−1^ MT+200 µmol L^−1^ Cd.

**Fig. S3.**

table 1. The transcriptomic data

| Sample | Raw Reads | Clean Reads | Reads mapped | Unique mapped | Q30(%) | GC Content(%) |
| --- | --- | --- | --- | --- | --- | --- |
| CK | 47289764 | 44946808 | 43997570(97.89%) | 41532703(92.40%) | 94.21 | 44.18 |
| CK | 47653854 | 44770634 | 43714487(97.64%) | 41309212(92.27%) | 94.37 | 43.11 |
| CK | 46340068 | 43140430 | 42148376(97.70%) | 39751540(92.14%) | 94.47 | 43.00 |
| T1 | 47011592 | 44386602 | 43526099(98.06%) | 41200883(92.82%) | 94.54 | 43.26 |
| T1 | 46983914 | 44837248 | 43932854(97.98%) | 41701156(93.01%) | 94.25 | 43.53 |
| T1 | 48235508 | 45103580 | 44189133(97.97%) | 41878862(92.85%) | 94.29 | 43.21 |
| T2 | 45800638 | 42886548 | 42020228(97.98%) | 39907892(93.05%) | 94.32 | 43.80 |
| T2 | 44177008 | 42889560 | 41986446(97.89%) | 39935908(93.11%) | 94.43 | 43.59 |
| T2 | 46228762 | 44696862 | 43791050(97.97%) | 41587321(93.04%) | 94.59 | 43.52 |
| T3 | 46986812 | 45683538 | 44682360(97.81%) | 42565558(93.17%) | 94.04 | 43.72 |
| T3 | 48641718 | 47343500 | 46377371(97.96%) | 44079038(93.10%) | 94.08 | 43.64 |
| T3 | 46760808 | 45238090 | 44274819(97.87%) | 42199431(93.28%) | 94.25 | 43.64 |

**Fig. S4.** The DEGs and DAMs involved in the pathway of valine, leucine and isoleucine degradation in response to Cd stress and the addition of MT.


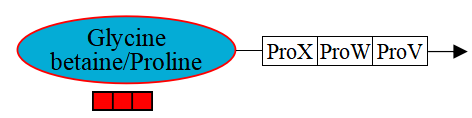

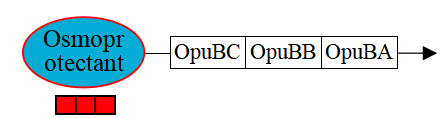

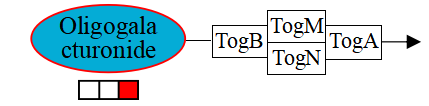

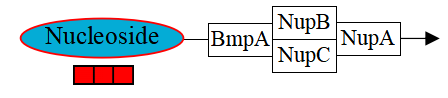

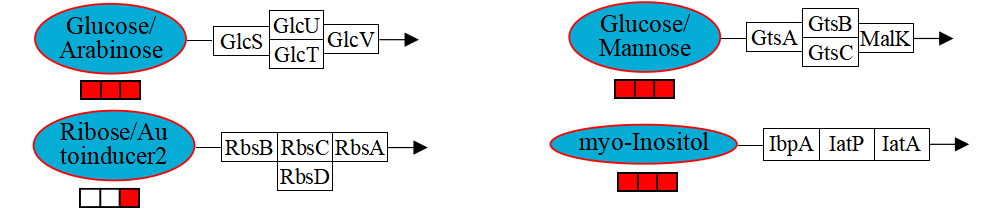

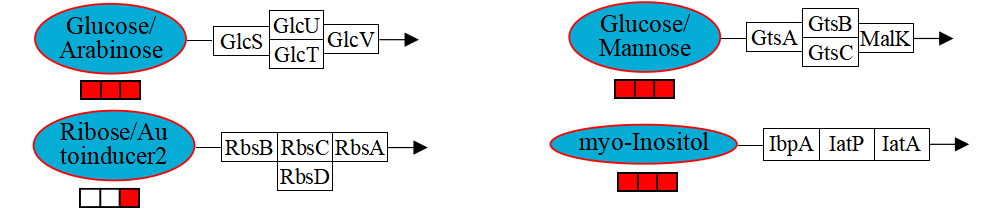

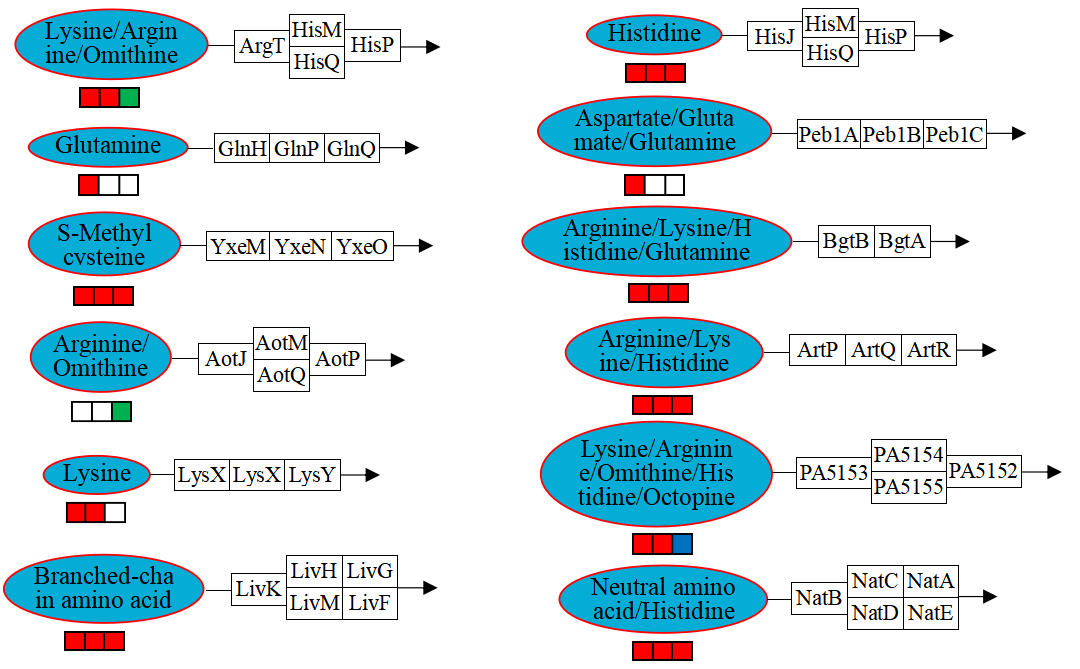

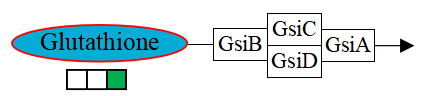

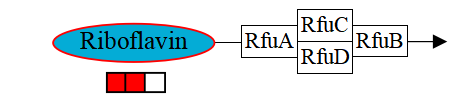

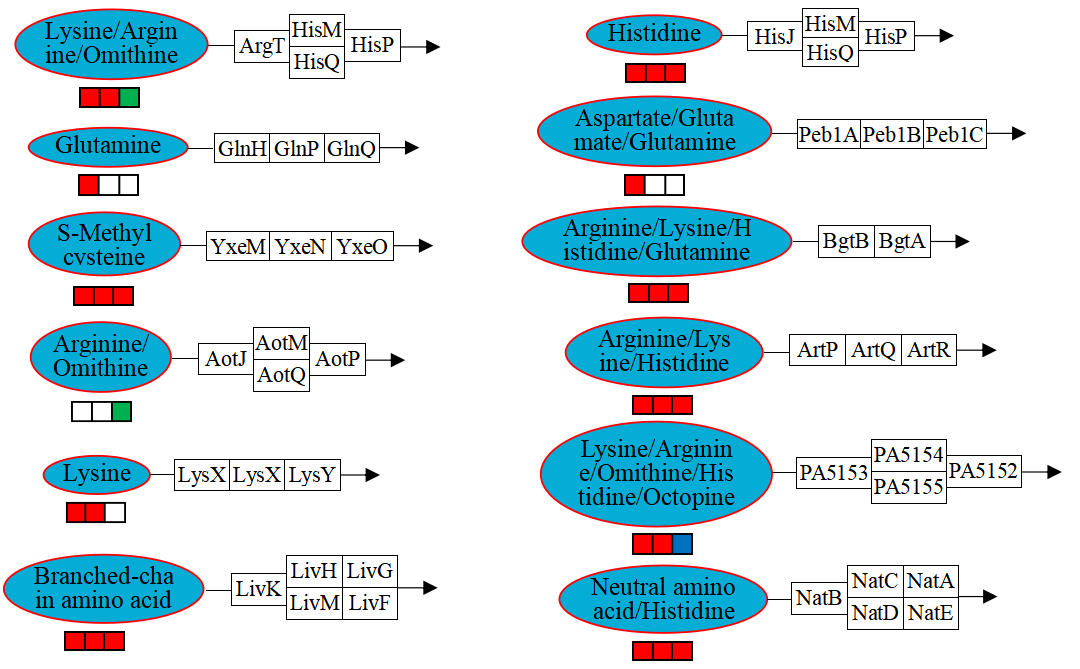

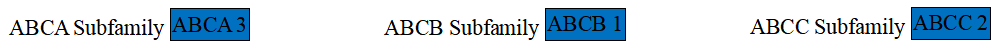

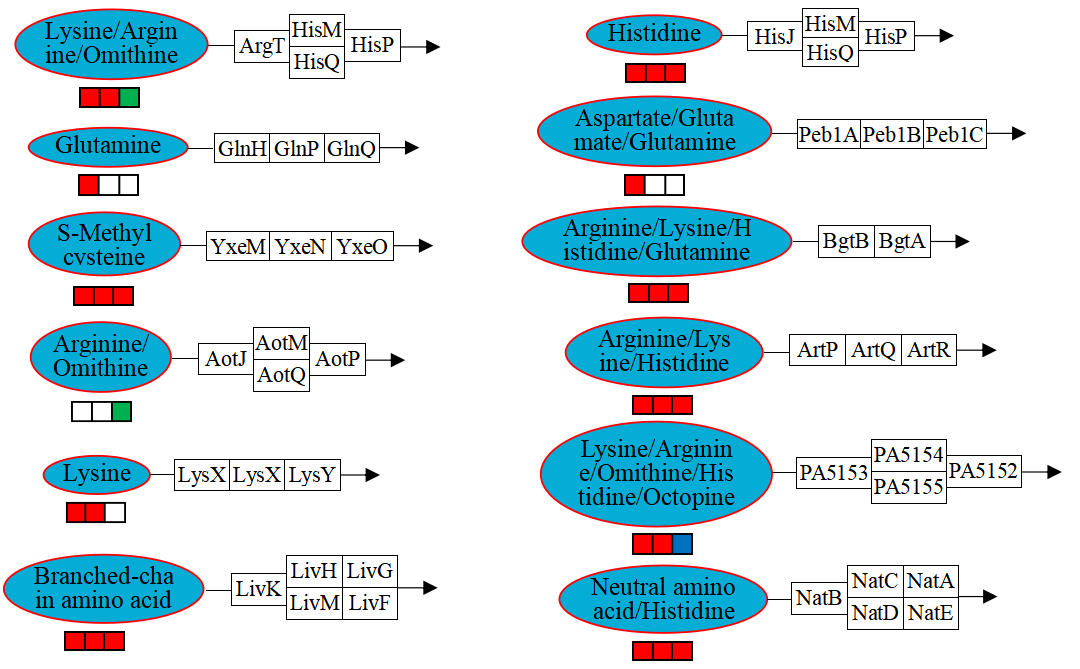

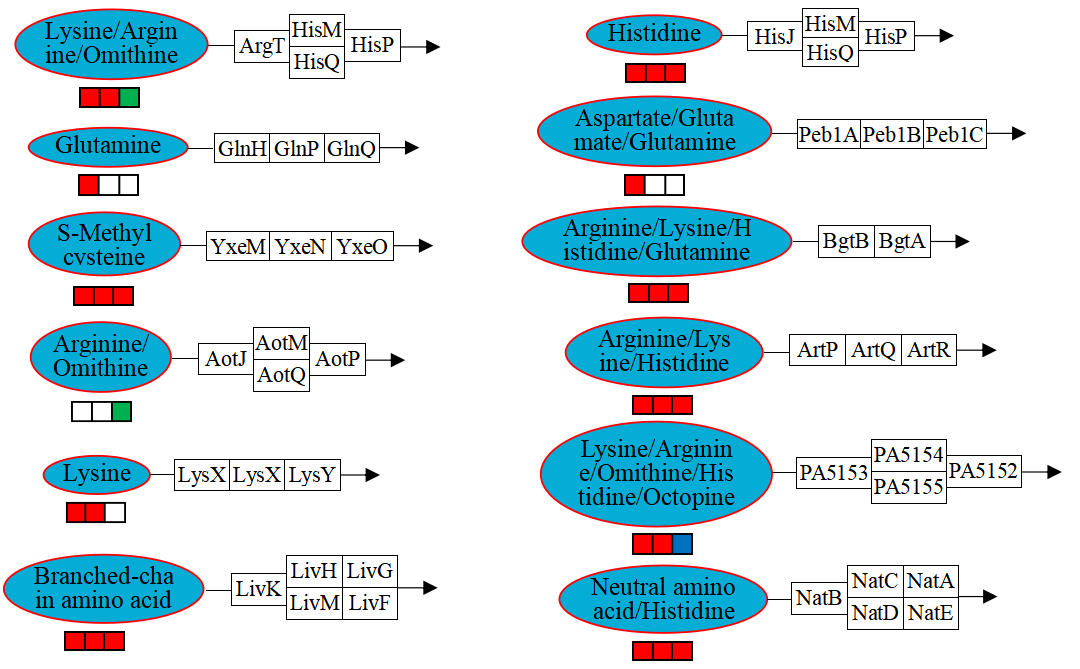

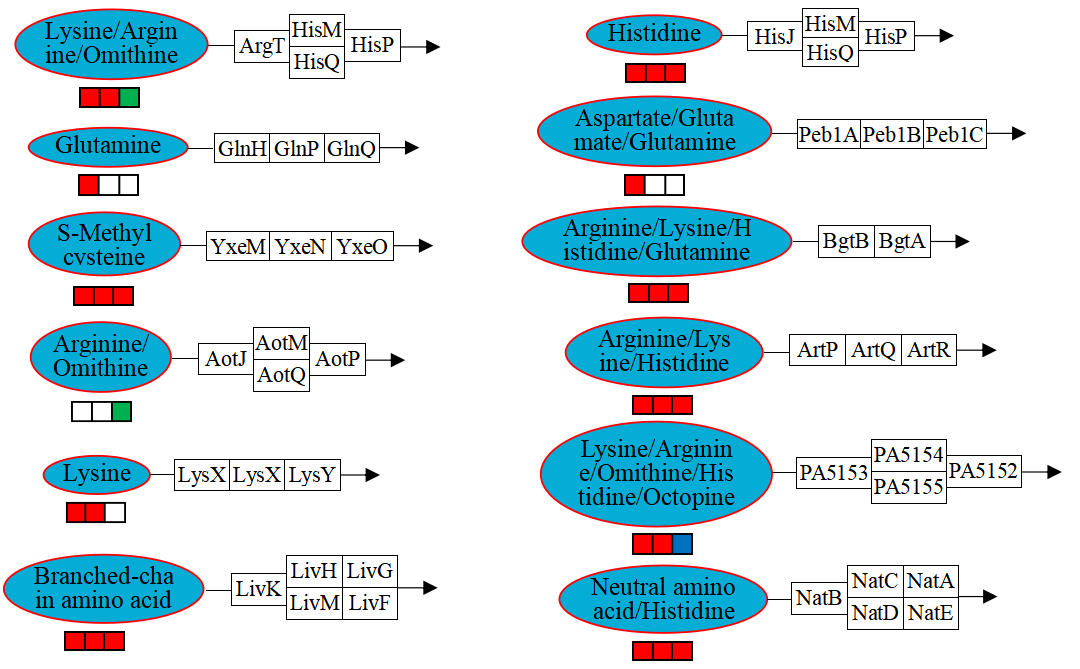

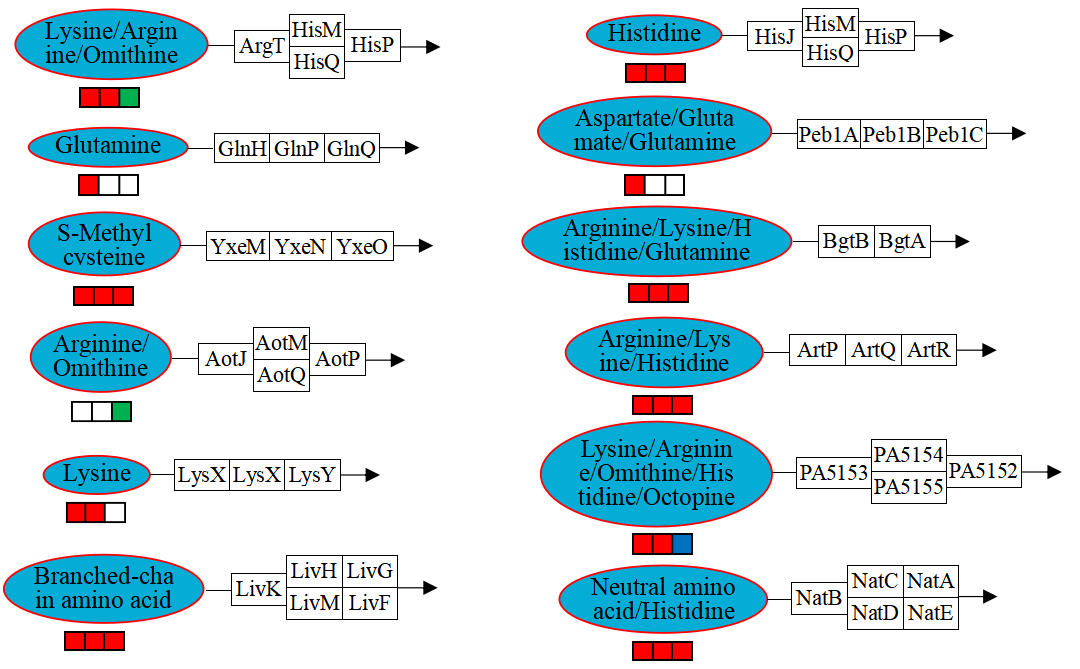

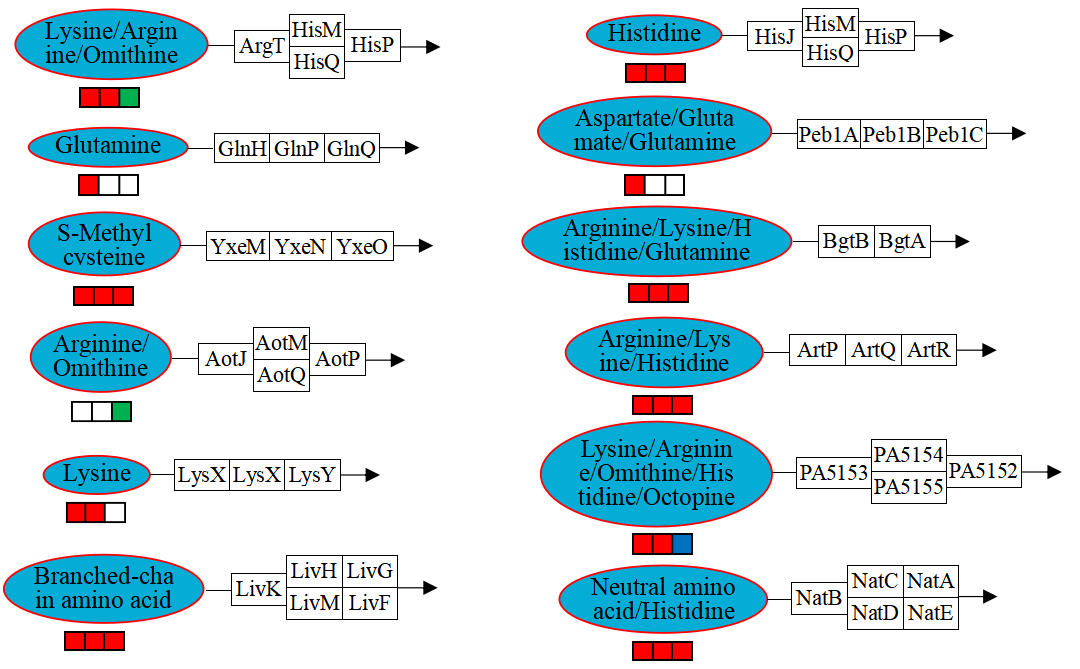

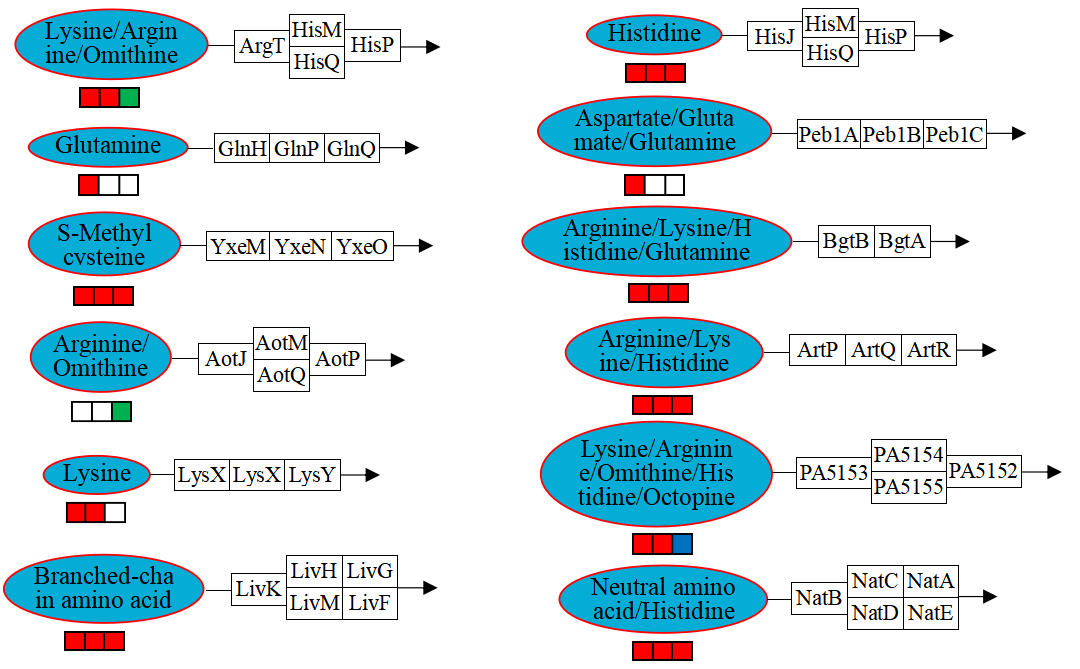

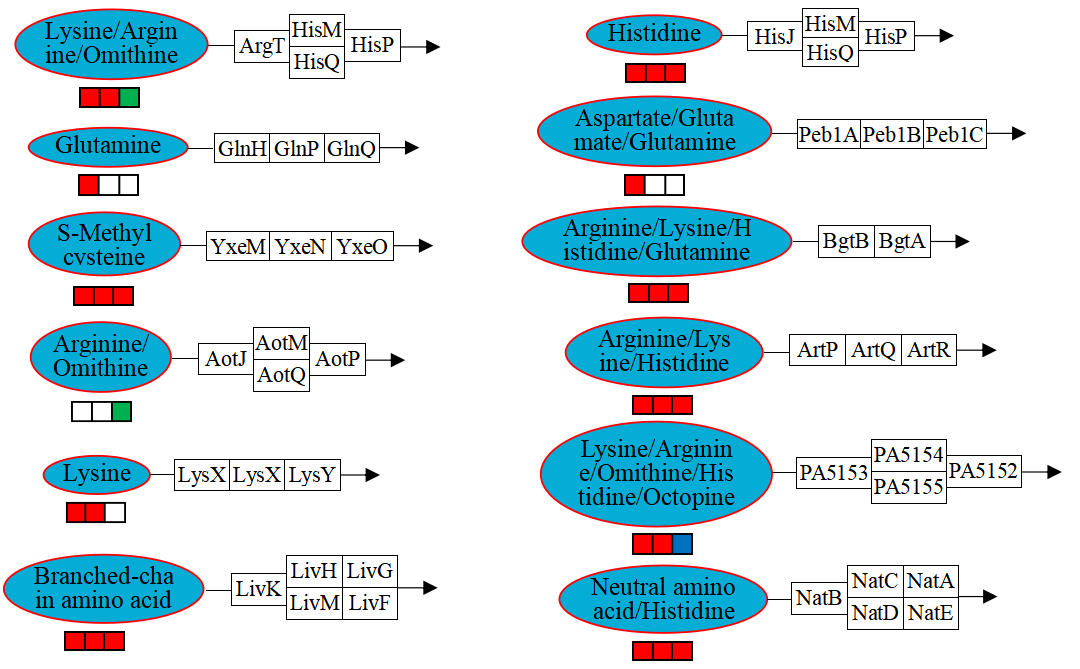

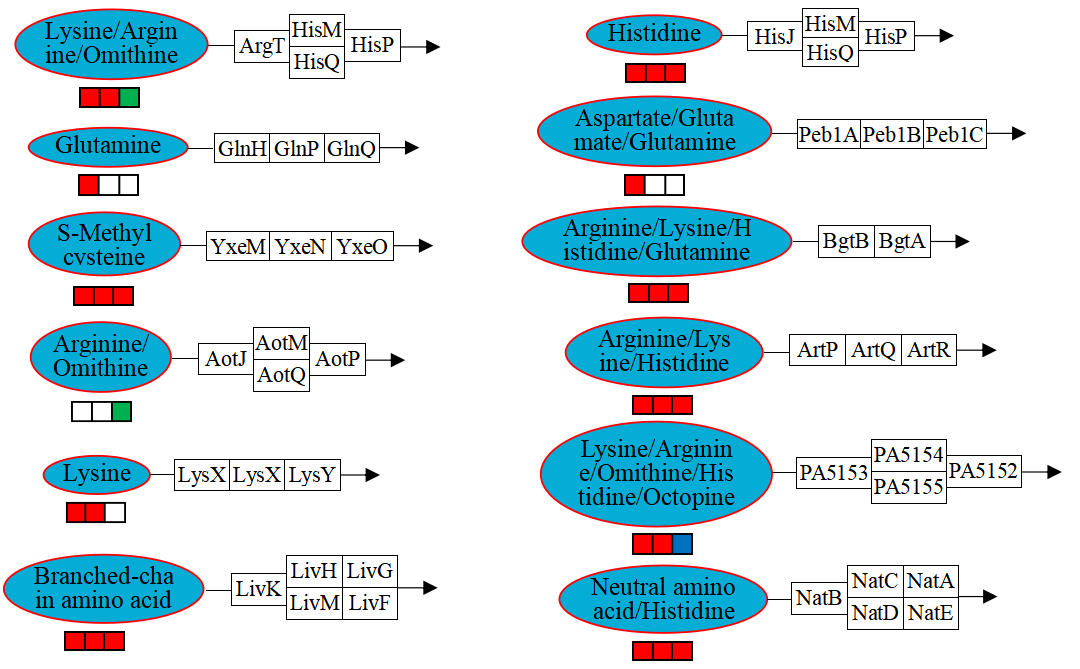

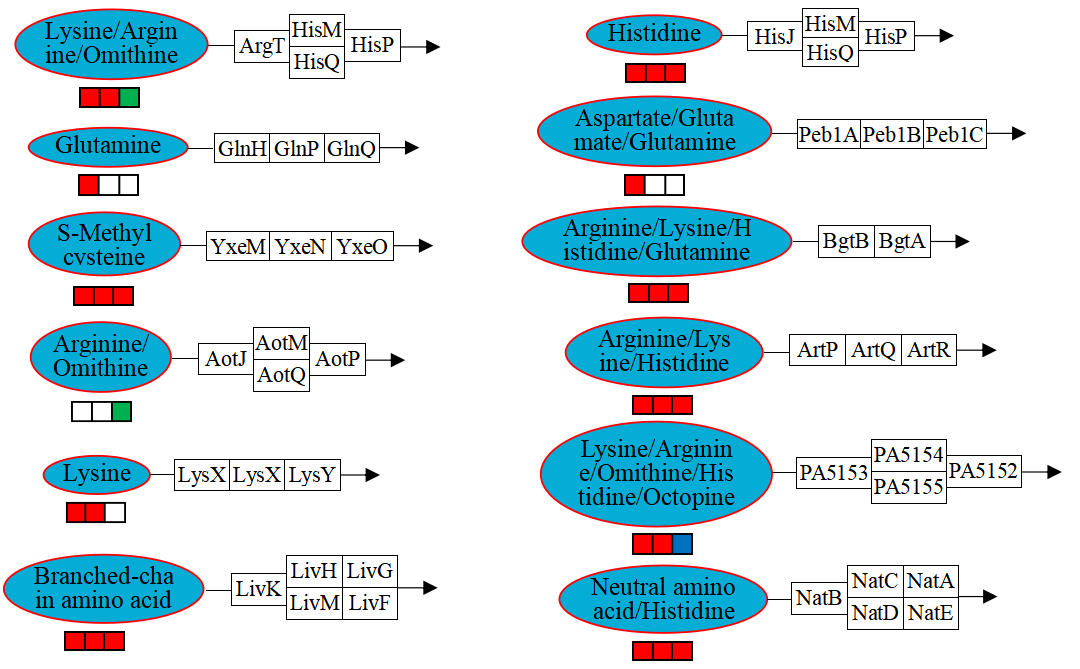

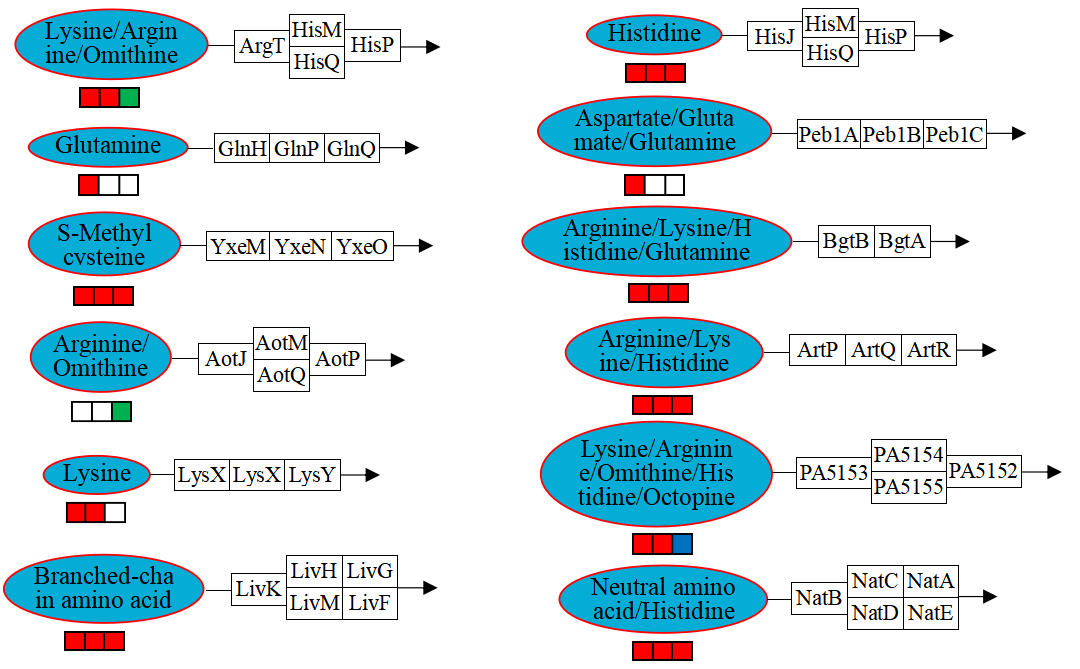

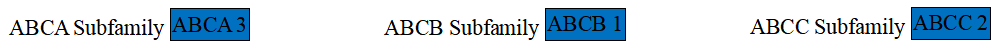

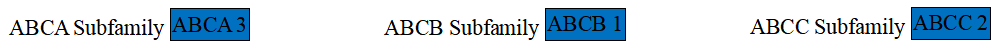


**A**

**B**

**C**

**D**

**E**

**F**

**G**

Note: A-F, Prokaryotic--type ABC transporters. A, Mineral and organic ion transporters; B, Oligosaccharide, polyol, and lipid transporters; C, Monosaccharide transporters; D, Phosphate and amino acid transporters; E, Peptide and nickel transporters; F, Metallic cation, iron-siderop hore and vitamin B12 transporters. G, different subfamily in eukaryotic-type ABC transporters. The blue pattern represents the DEGs or DEGs that changed under Cd stress and the addition of MT. The rectangle is divided into three equal parts (the left of rectangle represents DEGs or DEMs in CK vs T1, the middle of rectangle represents DEGs or DEMs in CK vs T2, the right of rectangle represents DEGs or DEMs in CK vs T3). The color in the rectangle represents the DEGs or DEMs are regulated under Cd stress and the addition of MT (red indicates up-regulation, green indicates down-regulation, blue indicates both up-regulation and down-regulation, white indicates neither up-regulation nor down-regulation).
